# Evaluation of software technical quality for collecting data from patients under palliative care

**DOI:** 10.1590/0034-7167-2023-0435

**Published:** 2024-07-29

**Authors:** Jéssica de Fátima Gomes Pereira, Letícia Pontes, Mitzy Tannia Reichembach Danski, Solena Ziemer Kusma Fidalski, Juliana de Oliveira dos Santos, Maria Gorete de Brito Cunha, Mairla Cristina Silva Mota, Edmilson Bezerra Cruz

**Affiliations:** I Universidade Federal do Paraná, Complexo Hospital de Clínicas, Empresa Brasileira de Serviços Hospitalares. Curitiba, Paraná, Brazil; II Universidade Federal do Paraná. Curitiba, Paraná, Brazil

**Keywords:** Palliative Care, Software, Data Collection, Nursing Process, Technology Assessment, Biomedical, Cuidados Paliativos, Programas Informáticos, Recolección de Datos, Proceso de Enfermería, Evaluación de la Tecnología Biomédica

## Abstract

**Objectives:**

to evaluate software technical quality for collecting data from patients under palliative care.

**Methods:**

this is methodological technology evaluation research, according to the technical standard International Organization for Standardization/International Electrotechnical Commission 25040-2011, developed from August 2021 to August 2023. Eight nurses and eight information technology professionals participated as judges, who evaluated six quality characteristics and 23 subcharacteristics. Items that reached a percentage of agreement greater than 70% were considered suitable.

**Results:**

the characteristics evaluated by nurses/information technology professionals received the following percentages of agreement, respectively: functional suitability (94%-84%); reliability (100-70%); usability (89.9-66.8%); performance efficiency (95.8%-86.1%); compatibility (95.8-79.6%); and safety (96%-83.4%).

**Conclusions:**

the software was considered suitable in quality evaluation to offer support to nurses in collecting patient data under palliative care, with the potential to operationalize the first Nursing Process stage.

## RESUMEN

**Objetivos:**

evaluar la calidad técnica del software para la recolección de datos de pacientes en cuidados paliativos.

**Métodos:**

se trata de una investigación metodológica de evaluación de tecnología, según la norma técnica *International Organization for Standardization/International Electrotechnical Comission* 25040-2011, desarrollada de agosto de 2021 a agosto de 2023. Participaron como jueces ocho enfermeros y ocho profesionales de tecnologías de la información, quienes evaluaron seis características de calidad y 23 subcaracterísticas. Se consideraron adecuados los ítems que alcanzaron un porcentaje de acuerdo superior al 70%.

**Resultados:**

las características evaluadas por enfermeros/profesionales de tecnologías de la información recibieron los siguientes porcentajes de acuerdo, respectivamente: adecuación funcional (94%-84%); confiabilidad (100-70%); usabilidad (89,9-66,8%); eficiencia del desempeño (95,8%-86,1%); compatibilidad (95,8-79,6%); y seguridad (96%-83,4%).

**Conclusiones:**

el *software* fue considerado adecuado en la evaluación de calidad para ofrecer apoyo a los enfermeros en la recolección de datos de los pacientes en cuidados paliativos, con potencial para operacionalizar la primera etapa del Proceso de Enfermería.

## INTRODUCTION

The World Health Organization (WHO) defines Palliative Care (PC) as an approach that improves the quality of life of patients, adults and children who experience problems associated with potentially fatal illnesses. This care, in turn, involves patients and family and is characterized by actions that seek to integrate emotional, social, spiritual and cultural aspects^(1)^.

When considering the dimension of this care, determining how nurses should assist patients under PC is complex and challenging, as care actions must be supported by a methodological work instrument, such as the Nursing Process (NP), which will allow for a broad evaluation and systematized care. In PC, the first NP stage is related, especially, to the identification of signs and symptoms. Therefore, data collection is the basis for developing an individualized and effective care plan that guarantees comfort and a dignified death^(2)^.

To improve PC data collection, especially in the identification and management of signs and symptoms, healthcare professionals can use technologies, such as Information and Communication Technologies (ICT). In the health area, ICT have become relevant strategies in knowledge management, mainly due to information digitalization. In this way, healthcare professionals incorporate this type of technology to improve assistance and scientific research production^(3)^.

As an example of ICT used in the healthcare sector, there are applications for mobile devices, which support patients and healthcare professionals in providing care. As an example, there is the point-of-care application, developed in Australia to promote adherence to good practice recommendations for managing symptoms of neurocognitive disorders^(4)^, the NeMo system, a mobile tool for home identification of neonatal diseases in Uganda^(5)^, and the *Meu PICC* application, developed in São Paulo for out-of-hospital monitoring of patients using peripherally inserted central catheters^(6)^.

However, when observing in clinical practice the need for technology to support the operationalization of the first NP stage, i.e., the collection of data from patients under PC, between 2017 and 2019, a mobile application-type software called *AVALIA TIS - Cuidados Paliativos*, which aims to support the collection of data from patients under PC. This application is based on the Theory of Basic Human Needs and palliative philosophy, which seek comfort as objective of care^(7)^.

However, it is considered that, for security in software use, it is necessary to evaluate quality, which is defined by regulations with technical specifications that define rules and criteria to ensure that software products are suitable for their intended use. These standards are essential, as they guide software product development and evaluation with the aim of meeting the expectations and quality required by customers^(8)^. Therefore, to continue the research that resulted in *AVALIA TIS - Cuidados Paliativos* development, it was decided to develop a technical quality evaluation study for this mobile technology.

It is understood that, to guarantee security in software use, technical quality evaluation is necessary to identify whether the technology meets users’ needs in the short, medium and long term, in addition to providing efficiency and safety in use, with adequate costs, productivity and functionality, allowing use with few or no defects^(9)^.

It is noteworthy that *AVALIA TIS - Cuidados Paliativos* is the product of a professional master’s degree dissertation in nursing from the *Universidade Federal do Paraná* (UFPR) to support nurses in collecting data from patients under PC. The application also strengthens the record of the first NP stage, by organizing patient data systematically in a document in Portable Document Format (PDF), which can be attached to the medical record^(7)^.

## OBJECTIVES

To evaluate the AVALIA TIS - Cuidados Paliativos mobile application technical quality, developed to support nurses in collecting data from patients under PC.

## METHODS

### Ethical aspects

This research is linked to a thematic project entitled “*Tecnologias para Qualificar e Consolidar a Sistematização da Assistência de Enfermagem nos Diferentes Cenários da Prática Profissional*”, approved by the Research Ethics Committee (REC) of the *Complexo Hospital de Clínicas* at UFPR (CHC-UFPR). The judges who participated in the software quality evaluation were asked to sign the Informed Consent Form (ICF).

### Study design, place and period

This is methodological technology evaluation research, in accordance with the International Organization for Standardization (ISO)/International Electrotechnical Commission (IEC) 25040-2011 technical standard^(10)^. The research was developed through the UFPR Graduate Program in Nursing, in Curitiba, Paraná, Brazil, from August 2021 to August 2023.

### Population or sample; inclusion and exclusion criteria

Two groups of judges made up of nurses and information technology (IT) professionals were obtained to evaluate the software. To define the sample, ISO/IEC-25062 standard recommendations were used, which indicates a minimum sampling of eight evaluators to guarantee the representativeness of the sample^(11)^.

The selection of professionals to compose the group of judges occurred through snowball sampling^(12)^. Inclusion criteria for the evaluators were being a nurse with academic and/or practical experience under PC, being an IT professional with academic and/or practical experience in developing mobile applications. Exclusion criterion was being evaluators who did not respond to the instrument within the given time.

**Figure 1:**
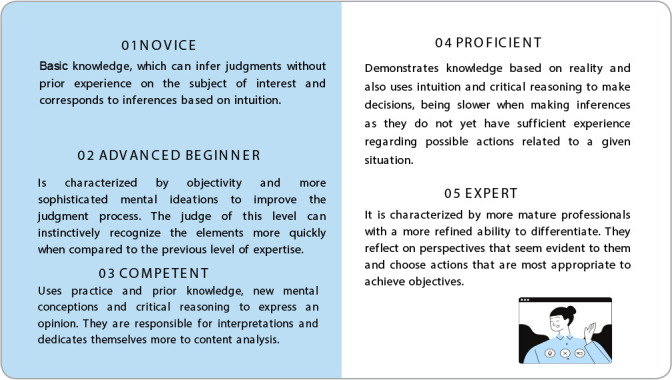
Classified according to level of expertise, Curitiba, Paraná, Brazil, 2023

*Note: adapted from BENNER; TANNER; CHESLA (2009).*

Judges were classified according to their level of expertise. In this classification, the level of knowledge is divided into five levels, ranging from novice to expert, described in Figure 1 ^(13)^.

**Figure 2:**
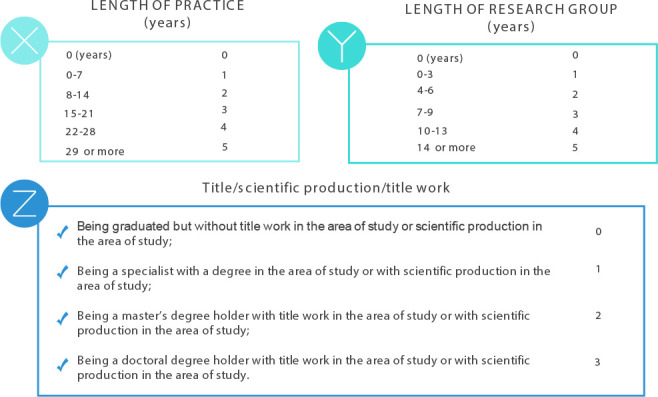
Classification according to the level of practical and academic experience, Curitiba, Paraná, Brazil, 2023

*Note: adapted from BENNER; TANNER; CHESLA (2009).*

To establish the level of expertise, the formula N = (X + Y + Z)/3 was used, where X corresponds to length of practice, Y, to length of research group, and Z, to scientific knowledge (title, title work and scientific production in PC) (Figure 2)^(13)^. It should be noted that the classification was carried out for leveling purposes and was not used as an inclusion and exclusion criterion.

### Study protocol

To evaluate the software, the ISO/IEC 25040-2011 software quality evaluation process was adopted, which consists of five stages^(10)^:

#### Stage I - Establish the evaluation requirements

**Figure 3:**
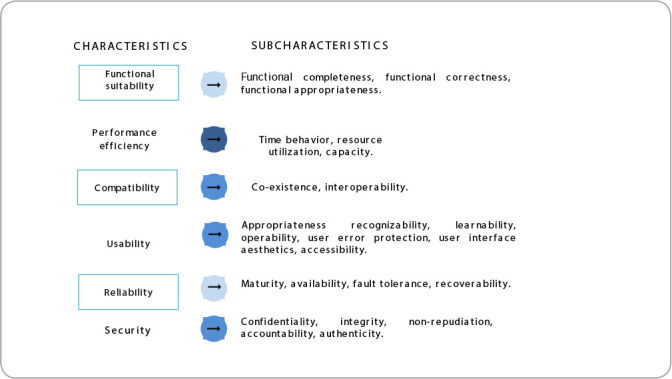
Product Quality Model: characteristics and subcharacteristics, Curitiba, Paraná, Brazil, 2023

*Note: adapted from ABNT ISO/IEC 25010 (2011).*

To establish the software evaluation requirements, we opted for the Product Quality Model, defined by ISO/IEC 25010-2011, which establishes eight characteristics and respective subcharacteristics that must be considered in evaluation: functional suitability; performance efficiency; compatibility; usability; reliability; security; maintainability; and portability^(14)^. It should be noted that, due to the impossibility of making the software source code available to evaluators, for reasons of technology security, it was determined to evaluate six of the eight quality characteristics, excluding maintainability and portability (Figure 3).

#### Stage II - Specify the evaluation

At this stage, quality metrics, scoring levels and criteria for judgment were defined. In the quality evaluation process, two forms were used to collect data regarding the software; these forms have already been validated for clarity, understanding and objectivity in a previous study^(15)^. To carry out this research, the forms were adapted and sent to the group of nurses and the group of IT specialists, containing questions regarding identification data and information related to quality characteristics and subcharacteristics. These instruments had scoring levels based on ABNT NBR ISO/IEC 14598-6 *Anexo C*: (C) Agree; (D) Disagree; (NA) Not Applicable; and Comments. Level C means the item meets the quality requirement; level D means that the item does not meet the quality requirement; and the level NA refers to the item that does not apply or was not evaluated^(8)^.

Questions that the evaluators were unable to evaluate, whether due to lack of information, resources or specific knowledge, marked with the “NA” option, were discarded. These answers do not score and, therefore, do not affect the evaluation.

**Figure 4:**
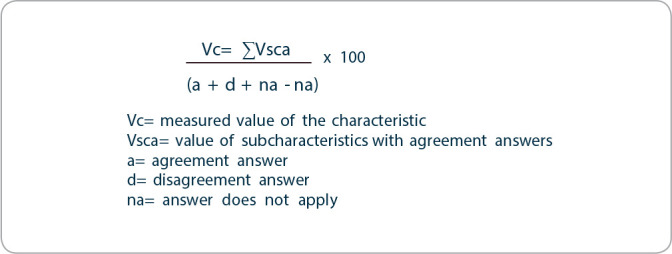
Formula for calculating percentages of quality characteristics and subcharacteristics, Curitiba, Paraná, Brazil, 2023

*Note: adapted from Oliveira and Peres (2015).*

The values in percentages of evaluated quality characteristics were obtained using the mathematical formula described in Figure 4.

Items with a percentage of agreement greater than 70% were considered suitable, according to the evaluation scale for subcharacteristics, adapted from ABNT NBR ISO/IEC 14598-6 *Anexo* C (Newsletter) (2004), in that 25% refers to poor, 50% to fair, 75% to good, and 100% to excellent^(8,15)^.

#### Stage III - Design the evaluation

This stage was characterized by planning activities and selecting participants to evaluate the software. Data collection took place electronically, with an invitation letter being sent to potential research participants, from September 2022 to January 2023. Those who agreed to participate in the research were asked to read and sign the ICF. They also received information about the application and the evaluation process of the quality characteristics to be evaluated, in addition to the link to download the application and the link to access the Google Forms^®^ software quality evaluation form. The test application sent to participants presents technical specifications compatible only with the Android system and, therefore, cannot be used on devices with the iOS system.

Participants had ten days to return the completed form; however, it was necessary to extend the deadline for another ten days in order to ensure that the evaluators completed the process. During this period, the researcher was available to answer questions and assist participants via email, telephone and WhatsApp^®^.

#### Stage IV - Execute the evaluation

Data were collected and tabulated in Microsoft Excel^®^ spreadsheets and analyzed using the Statistical Package for the Social Sciences (SPSS^®^) (IBM^®^ SPSS^®^ Statistics v. 25.0, SPSS Inc, Chicago, USA). The results were expressed as means, medians, minimum values, maximum values and standard deviations (quantitative variables) or as frequencies and percentages (qualitative variables). To carry out this stage, we opted for the help of statistics.

#### Stage V - Conclude the evaluation

Data analysis aimed to conclude whether *AVALIA TIS - Cuidados Paliativos* presents technical quality for most of the characteristics and subcharacteristics evaluated. The data is presented in table format.

## RESULTS

A total of 16 judges participated, eight nurses and eight IT professionals. Among nurses, females prevailed, with seven women (87.5%) and one man (12.5%) aged between 28 and 62 years. In relation to degrees, five (62.5%) held a master’s and three (37.5%) were specialists. Regarding the level of expertise, three were classified (37.5%) as novice, three (37.5%) as advanced beginner, and two (25%) as competent.

As for IT professional judges, the majority were male, six men (75%) and two women (25%), aged 23 and 46. Of these, six (75%) were specialists and two (25%) were doctors. The level of expertise was classified as five (62.5%) novice, one (12.5%) advanced beginner, one (12.5%) competent and one (12.5%) proficient.

Snowball sampling for judge selection allowed evaluating the application by professionals from four regions of Brazil (South, Southeast, North, Northeast) and a judge from Portugal, allowing evaluating the technology from different perspectives. Table 1 presents the results of software evaluation using the instruments used.

**Table 1:** Distribution of values according to the quality subcharacteristics and characteristics of *AVALIA TIS - Cuidados Paliativos* by nurses and information technology specialists, Curitiba, Paraná, Brazil, 2023 (N=16)

Variables	Nurse	IT
	n = 8	n = 8
	VC (%)	VC (%)
Functional completeness	100.0	93.8
Functional correctness	95.8	83.3
Functional appropriateness	87.5	75.0
Functional suitability Maturity	94.4 100.0	84.0 62.5^*^
Fault tolerance	100.0	87.5
Recoverability	100.0	42.9^*^
Availability	100.0	87.5
Reliability Appropriateness recognizability	100.0 93.8	70.1 65.6^*^
Learnability	95.8	70.8
Operability	100.0	75.0
Accessibility	50.0	33.3^*^
User error protection	100.0	87.5
User interface aesthetics	100.0	68.8^*^
Usability Time behavior	89.9 100.0	66.8^*^ 87.5
Resource utilization	87.5	87.5
Capacity	100.0	83.3
Performance efficiency Interoperability	95.8 91.7	86.1 79.2
Co-existence	100.0	80.0
Compatibility Confidentiality	95.8 100.0	79.6 87.5
Integrity	80.0	86.6
Non-repudiation	100.0	85.7
Accountability	100.0	71.4
Authenticity	100.0	85.7
Security	96.0	83.4

Source: authors (2023). VC - measured value of the characteristic; % - value in percentage; IT - information technology specialists;

* VC value < 70%.

In functional suitability evaluation, it was observed that, in the functional completeness subcharacteristic, all nurse judges agreed that the application meets the capacity to support the evaluation of patients under PC. As for the reliability characteristic, the maturity and recoverability subcharacteristics received an evaluation below 70% by IT judges. They suggested improving the application’s ability to save registered information before completing the data collection process. One of the judges, coded with the letter T, followed by numbering, in evaluating this characteristic, warned that the application:

*There is no routine for resuming filling in the event of a process interruption.* (T1) - (recoverability)

The usability characteristic was the only characteristic that presented an average evaluation below 70% by IT judges. For the appropriateness recognizability, learnability, user error protection and user interface aesthetics subcharacteristics, judges pointed out that:

*In the questionnaire phases, always at the beginning, there is a brief explanation about the objective of the questionnaire. There is a scale about pain and what each level of the scale represents.* (T4) - (appropriateness recognizability and suitability)

*Fields with scales must have suggested filling values, and navigation between fields is only by selecting the field.* (T1) - (learnability)

*In the fields where you need to enter data that is numbers, it does not allow letters to be entered.* (T5) - (user error protection)

*It has a lot of potential to improve. I believe that the menu would look better if it were centralized.* (T5) - (user interface aesthetics)

The usability characteristic also received comments from nurse judges, especially in the appropriateness recognizability, accessibility and user interface aesthetics subcharacteristics. Nurse judges were coded with the letter N, followed by numbering:

*Application is self-explanatory.* (N5) - (appropriateness recognizability)

*I was unable to identify support for use by people with disabilities.* (N5) - (accessibility)

*The possibility of changing the color could be offered if the user wishes.* (N3) - (user interface aesthetics)

In the performance efficiency and compatibility characteristics, all subcharacteristics received an evaluation above 70%, with emphasis on response time and interoperability of *AVALIA TIS - Cuidados Paliativos*:

*Its touch response time was very good.* (T1) - (time behavior)

*Suitable storage capacity.* (T7) - (capacity)

*Interaction is good for exchanging information effectively and efficiently.* (T7) - (interoperability)

*The time is a little long on the first access.* (N3) - (time behavior)

*Nursing evaluation provides a summary of everything that was evaluated in the application modules.* (N5) - (interoperability)

The sixth and last characteristic evaluated, security, had four subcharacteristics evaluated with a percentage above 70% by both groups of judges, who consider that the application is safe for use in clinical practice:

*I tried to enter another email, but it didn’t work.* (T5) - (integrity)

*Registration required, including identification of the COREN number and authorization to use the application.* (N5) - (authenticity)

## DISCUSSION

Using assistance technologies to support the operationalization of NP is a fact, being in vast development in the Brazilian reality, which indicates progressive computerization of nursing. Using these digital resources as support for nurses serves to enhance, qualify and effectively provide care^(16)^.

In this study, six characteristics and 23 subcharacteristics relating to *AVALIA TIS - Cuidados Paliativos* quality were evaluated. Of the six characteristics evaluated, only usability presented an evaluation percentage below 70% by IT professional judges, a fact that did not prevent the evaluation by nurse judges, who considered this characteristic suitable.

Nurse judges considered that the application is 89.9% suitable, and this reflects the application’s capacity to support nurses in carrying out data collection specific to patients under PC. The judges also consider that the application is self-explanatory, a fact that makes the technology easy for users to use.

However, it is possible to observe some suggestions to improve application usability, such as the need for a more dynamic menu to facilitate patient evaluation. It is noteworthy that this aspect was planned at the beginning of the technology’s development; however, due to financial limitations, some specifications were not included in the first version. Furthermore, in the usability characteristic, the accessibility subcharacteristic received a score below the expected average. This aspect was not thought of in *AVALIA TIS - Cuidados Paliativos* development, being a subcharacteristic that should be considered in a next version of the application.

As benefits evidenced in *AVALIA TIS - Cuidados Paliativos* evaluation, it was observed that this software is easy to access, as it is a mobile application that allows nurses to evaluate patients at the bedside. It is understood that, with the advancement of cell phones, the scope of applications for smartphones has expanded, with the number of applications developed for use in the clinical evaluation of patients increasing^(17)^.

Using high-quality applications in the health sector can be responsible for gathering information necessary for the emergence of new discoveries for clinical practice, in addition to linking information to improve assistance and radically modify patient care^(3)^. In this context, in Switzerland, in 2019, an application was developed and evaluated to support bedside nursing care. This application has several features that allow professionals to identify patient data, such as enabling access to medical records by reading the code present on patient identification bracelets using the smartphone’s camera^(18)^.

The performance efficiency characteristic of *AVALIA TIS - Cuidados Paliativos* received a good evaluation from judges, with emphasis on the application’s response time and storage capacity. As the central idea of the technology is to offer nurses support to carry out a robust clinical evaluation that meets all dimensions of patients under PC, the application is extensive, requiring some access for users to adapt to the interfaces.

To clinically evaluate patients under PC, it is necessary to understand that this care was created to treat the person as a whole and not just the disease. In this way, the prioritization of typically peripheral domains of functioning, such as psychosocial, spiritual and existential, becomes fundamental. The general objective of this care is to obtain the best possible quality of life^(19)^.

In the context of mobile applications used in health, *AVALIA TIS - Cuidados Paliativos* has similar functionality to the *CuidarTech Semio - Exame Clínico de Enfermagem* application, as, in addition to being a technological innovation, both are supported by the Theory of Basic Human Needs, serving as an educational and professional support tool that helps students and nurses in carrying out data collection, promoting scientific knowledge and clinical reasoning^(20)^.

Another benefit found by judges in technical quality evaluation was the interoperability characteristic, due to the application’s ability to generate a summary of all data collected in clinical evaluation, guaranteeing the recording of the first NP stage. It must be considered that recording the data collected by nurses in medical records is a form of multidisciplinary communication. This record can be carried out via traditional or electronic means, ensuring continuity and quality of care^(21)^.

In addition to the record generated, application security is a highlight in quality evaluation, involving the confidentiality of the data entered. To access the data, the software requires a professional to register, approved by an application administrator, who may be responsible for the unit where the technology will be used. The application also allows users to recover their access password, which facilitates technology use.

It is considered that the technical quality evaluation process used in this study is complex, as it involves a series of phases and steps so that quality characteristics are evaluated and allow it to be identified whether the technology is capable of meeting user needs. The benefits found in technical quality evaluation of *AVALIA TIS - Cuidados Paliativos* indicate that this is an innovative technology, as it is capable of guiding nurses to carry out detailed data collection from patients under PC, having a series of specific scales to measure signs and symptoms. This technology is easy to access, enabling nurses to have a detailed and reliable summary of the data collected and providing support for creating a care plan aimed at patients’ real needs.

### Study limitations

This research presented limitations related to the test application, as it is only compatible with the Android system, a situation that made it difficult for judges to accept data collection, in addition to the need for a patient care unit under PC to apply technology in clinical practice.

### Contributions to nursing

The technical quality evaluation process used in this study showed that *AVALIA TIS - Cuidados Paliativos* is a tool with the potential to be used both to support nurses in clinical practice and for health teaching, with the aim of directing undergraduate students’ gaze to individuals’ multidimensionality, in addition to being an instrument for collecting data in future research. Furthermore, it is understood that the method used in this research can serve as a basis for future studies that focus on evaluating software technical quality in healthcare. It is considered that this research is an advance for the area of nursing in terms of safe use of software for care practice.

## CONCLUSIONS

*AVALIA TIS - Cuidados Paliativos* quality is suitable for multidimensional evaluation of patients under PC, enabling nurses to advance in the other NP stages. When considering that the quality evaluation process of *AVALIA TIS - Cuidados Paliativos* involved a series of characteristics, this mobile technology presented good results, despite the below average evaluation in the usability characteristic by the group of IT judges.

The comments recorded show that the first version of the application is easy for nurses to access. However, there may be investments for improvements, such as the insertion of a menu that allows nurses to select the evaluation interface they want, without needing to go through all the screens. The application’s general objective, clinical evaluation, is recognized in judges’ evaluation, with emphasis on registration after the evaluation process, a benefit that guarantees the completion of the NP first stage.

As a suggestion for future research, it is recommended that *AVALIA TIS - Cuidados Paliativos* can be evaluated in nurses’ and nursing students’ clinical practice in order to verify its effectiveness for evaluating relevant aspects of patients under PC.

## Supplementary Material





## Data Availability

https://doi.org/10.48331/scielodata.CANHHY
