# Supplementary material for: Evaluation of software technical quality for collecting data from patients under palliative care
Source: Rev Bras Enferm. 2024 Jul 29;77(3):e20230435. doi: 10.1590/0034-7167-2023-0435 (PMC11290725; doi:10.1590/0034-7167-2023-0435)
Supplement: Supplementary file 1 [file 0034-7167-reben-77-03-e20230435-suppl01.pdf]

IDENTIFICAÇÃO DO PROJETO:

Título: Tecnologias para qualificar e consolidar a sistematização da assistência de enfermagem nos diferentes cenários da prática profissional.

Pesquisador Principal: Letícia Pontes

Colaboradores:

Professores –Mitzy Tannia Reichembach Danski; Márcia Helena de Souza Freire;
Mestrandos – Bruna Morelli Bottega; Jéssica de Fátima Gomes Pereira; Mariá Comparin Machado; Franciele Rocha; Fabíola Nascimento; Vanda Aparecida Tolari; Laryssa Sampaio Silva

Local de Realização: Complexo Hospital de Clínicas;

Instituição de Ensino: Universidade Federal do Paraná

Finalidade: Projeto para desenvolvimento no Programa de Mestrado Profissional

Período da Pesquisa: agosto de 2018 a fevereiro de 2019.

Instituição co-participante: Hospital Infantil Waldemar Monastier;

DESENHO DO ESTUDO

Trata-se de uma pesquisa exploratória a ser desenvolvida no complexo Hospital de Clinicas da UFPR, tendo como instituição co-participante o Hospital Infantil Waldemar Monastier.

Participarão do estudo, enfermeiros assistenciais que atuam nos três turnos de trabalho das Unidades de: Terapia Intensiva Adulto e Pediátrica; Clínica Médica e Cirúrgica do complexo Hospital de Clínicas da UFPR e, da Unidade de Terapia Intensiva Neonatal do Hospital Waldemar Monastier.

Esta pesquisa se desenvolverá em duas etapas, na primeira etapa se dará o desenvolvimento de uma Tecnologia Assistencial para a avaliação clínica do enfermeiro de pacientes em tratamento clínico, submetidos a procedimentos cirúrgicos, em cuidados paliativos, em cuidados intensivos e, de crianças com suspeita de morte encefálica. A segunda etapa terá como objetivo implantar o uso da Tecnologia Assistencial desenvolvida nas unidades de internação, campos desta pesquisa.

RESUMO

Trata-se de uma **pesquisa exploratória**, a ser desenvolvida no Complexo Hospital de Clínicas da UFP e no Hospital Infantil Waldemar Monastier, localizados na cidade de Curitiba e Campo Largo – Paraná, respectivamente. Tem a proposta de consolidar o uso do Processo de Enfermagem, considerado um instrumento ou modelo metodológico utilizado para favorecer o cuidado e organizar as condições necessárias para que ele aconteça. A utilização do Processo de Enfermagem contribui para a consolidação da Sistematização da Assistência de Enfermagem (SAE), caracterizada como uma ferramenta que auxilia na organização do trabalho e orientação da prática profissional da enfermagem, envolvendo aspectos que transcendem o cuidado direto. O Conselho Federal de Enfermagem considera que a materialização da SAE, tendo como um dos elementos o Processo de Enfermagem (PE), contribui efetivamente para a melhoria da qualidade de assistência em enfermagem. O planejamento realizado através do PE caracteriza-se por um processo

tecnológico capaz de orientar a sequência do raciocínio lógico, melhorando dessa forma a qualidade do cuidado por meio da sistematização da avaliação clínica, da identificação dos diagnósticos de enfermagem, da elaboração das intervenções e da monitorização dos resultados do cuidado de Enfermagem. O PE inclui diferentes fases para sua efetivação e, a primeira se caracteriza pela **avaliação clínica** do paciente, a qual permite ao enfermeiro interpretar e agrupar os dados que auxiliam na tomada de decisão, para o planejamento do cuidado. Tendo como questão de pesquisa – A implementação de uma tecnologia assistencial para avaliação clínica do enfermeiro, contribui para o uso do PE no planejamento do cuidado? – O **objetivo geral** desta pesquisa é desenvolver tecnologias assistenciais que auxilie o enfermeiro na avaliação clínica diária de pacientes hospitalizados. A hipótese é que o desenvolvimento de uma Tecnologia Assistencial para a Avaliação Clínica do Enfermeiro contribui para a efetiva utilização do Processo de Enfermagem no planejamento do cuidado e, consequentemente, na consolidação da SAE nas unidades de internação. A pesquisa será **desenvolvida** em duas etapas no Complexo Hospital de Clínicas (CHC) da UFPR e, terá como Instituição Co-participante o Hospital Infantil Waldemar Monastier. Os **campos de pesquisa** serão: Unidade de Clínica Médica; Unidade de Cirurgia Geral; Unidade de Terapia Intensiva Adulto e Unidade Pediátrica do CHC; Unidade de Terapia Intensiva Neonatal do Hospital Infantil Waldemar Monastier. Os **participantes** serão todos os enfermeiros assistenciais que atuam nos três turnos de trabalho das Unidades de: Terapia Intensiva Adulto e Pediátrica; Clínica Médica e Cirúrgica do complexo Hospital de Clínicas da UFPR e, da Unidade de Terapia Intensiva Neonatal do Hospital Infantil Waldemar Monastier. Como **critérios de inclusão** elencaram-se: atuar como enfermeiro assistencial nas Unidades campo da pesquisa; interesse em participar da discussão do tema da pesquisa; concordar em participar do estudo e assinar o Termo de Consentimento Livre e Esclarecido (TCLE). Como **critérios de exclusão**: estar afastado das atividades assistenciais no período de coleta de dados; declarar não haver interesse em participar da discussão do tema da pesquisa. A **primeira etapa** da pesquisa se refere ao desenvolvimento das tecnologias assistenciais e, os dados serão obtidos em duas fases, no período de agosto de 2018, logo após a aprovação do Comitê de Ética em Pesquisa, a fevereiro de 2019. Na **primeira fase da coleta de dados**, realizar-se-á: a)

busca bibliográfica, em bases de dados científicos, sobre o objeto de pesquisa, a saber – avaliação clínica e tecnologia assistencial –; **b)** busca de informações junto aos participantes da pesquisa, em relação a sua prática na avaliação clínica do paciente sob seus cuidados. Após a obtenção dos dados, se iniciará a construção da Tecnologia Assistencial proposta. Na **segunda fase,** os dados serão obtidos por meio de dois encontros entre pesquisador e participantes, que terão espaço para discussão sobre "Avaliação Clínica do Enfermeiro para a efetivação do Processo de Enfermagem"; Apresentação da Tecnologia Assistencial para avaliação clínica do enfermeiro aos participantes. Após apreciação da tecnologia, os participantes farão uma avaliação e apresentarão possíveis contribuições para o aprimoramento da tecnologia. Esses dados serão registrados em um diário de campo, para posterior análise, a partir da triangulação dos dados obtidos – informações dos participantes da sua prática clínica – dados da literatura. A **segunda etapa** da pesquisa terá como objetivo implantar a Tecnologia Assistencial desenvolvida. Acredita-se que o desenvolvimento de uma Tecnologia Assistencial para a avaliação clínica específica do enfermeiro, possa fornecer informações efetivas que tragam elementos para a elaboração de diagnósticos de enfermagem e a definição das melhores estratégias de intervenção, de maneira a consolidar a prática clínica da enfermagem, contribuir para o bom emprego do PE e materializar a SAE.

INTRODUÇÃO

A Sistematização da Assistência de Enfermagem (SAE) caracteriza-se como uma ferramenta que auxilia na organização do trabalho e orientação da prática profissional da enfermagem. Ela envolve aspectos que transcendem o cuidado direto, possibilitando a avaliação da eficiência e eficácia das atividades realizadas e, contribuindo para a tomada de decisão gerencial e política, com foco na excelência do cuidado (GUTIÉRREZ; MORAIS, 2017).

Carvalho e Bachion (2009) afirmam que a sistematização pressupõe a organização em um sistema, ou seja, um conjunto de elementos dinamicamente inter-relacionados. Para eles, na sistematização da assistência em saúde, esses elementos podem ser um conjunto de ações ou uma sequência de passos que busca um determinado fim como, por exemplo, os planos de cuidados, os protocolos e a padronização de procedimentos. A SAE oferece, ainda, suporte para operacionalizar o Processo de Enfermagem (PE).

O PE pode ser entendido como instrumento ou modelo metodológico utilizado para favorecer o cuidado e para organizar as condições necessárias para que ele aconteça. (GARCIA, TB; NOBREGA, MML; 2009).

No Brasil, a utilização do Processo de Enfermagem como um elemento que contribui para a Sistematização da Assistência de Enfermagem iniciou em 1978, com a publicação do livro “Processo de Enfermagem”, de autoria da professora Wanda de Aguiar Horta (HORTA, 1979). Mas, ainda nos dias de hoje, a incorporação do PE representa um desafio para as escolas e os serviços de saúde (BOTTOSSO, 2014).

O PE para Garcia (2016) deve ser o fundamento, o eixo estruturante da construção do conhecimento e, consequentemente, da prática profissional, já que o cuidado é o objeto de estudo e de trabalho da enfermagem. Já, Alfavaro-LeFevre (2014) consideram o PE um modelo de pensamento crítico essencial para promover um nível competente de cuidado e compor a base da tomada de decisão no processo assistencial. Vai além de orientar o planejamento formal dos cuidados e sua documentação. Deve orientar o pensamento dos enfermeiros, cotidianamente.

O Conselho Federal de Enfermagem (COFEN, 2009) conceitua PE como um instrumento metodológico que orienta o cuidado profissional de Enfermagem e a documentação da prática profissional, além de apoiar a contribuição da Enfermagem na atenção à saúde da população, conferindo maior visibilidade e reconhecimento profissional. Considera, ainda, que o PE deve estar baseado num suporte teórico que oriente a coleta de dados, o estabelecimento de diagnósticos e o planejamento das ações e, forneça a base para a avaliação dos resultados de enfermagem alcançados.

Portanto, não há dúvidas de que o PE é uma ferramenta indispensável para o enfermeiro determinar a ordem de prioridade das intervenções de enfermagem. Identifica, ainda, como os pacientes respondem aos cuidados prestados. Esse processo, ao gerar informações sobre o paciente, contribui para o planejamento da assistência, oferecendo dados do paciente a outros profissionais de saúde, por meio dos registros clínicos no prontuário (CLAUDINO et al, 2013).

O COFEN e, por conseqüência, o Conselho Regional de Enfermagem do Paraná (COREN-PR) e a Associação Brasileira de Enfermagem (ABEn), em suas diversas esferas administrativas, há cerca de duas décadas têm praticado esforços para a consolidação da SAE em todas as instituições de saúde brasileiras e, consequentemente, a utilização do PE para o planejamento de cuidado. Este Conselho profissional considera, ainda, que a materialização da SAE, tendo como um dos elementos o Processo de Enfermagem, contribui efetivamente para a melhoria da qualidade de assistência em enfermagem (COFEN, 2009).

No entanto, se tem evidenciado um desafio sua implantação. Diferentes fatores têm dificultado a adesão dos enfermeiros na utilização do Processo de Enfermagem na sua prática como, por exemplo, o número insuficiente de profissionais, agregado ao tempo minimizado para o cuidado, a falta de ações educativas nos serviços, a inadequação de instrumento para avaliação e registros e os recursos escassos. A ausência de padronização da linguagem, considerada essencial para o funcionamento da SAE (MARINELLI et al., 2016) também tem sido apontada como um complicador para a utilização do PE e, por que não dizer, a ausência de um modelo de avaliação clínica do enfermeiro, que atenda as necessidades do paciente, mas, também, que seja possível sua realização por esse profissional, considerando as características do seu processo de trabalho.

No campo em que se realizará a proposta, na maioria das unidades, o enfermeiro utiliza-se de um modelo de avaliação clínica adaptada do modelo médico. O modelo de avaliação clínica do profissional médico, já consolidado, atende as características do seu processo de trabalho, diferente do processo de trabalho do enfermeiro.

O PE inclui diferentes fases para sua efetivação e, a primeira se caracteriza pela avaliação clínica do paciente, a qual permite a interpretação e o agrupamento dos dados que auxiliam na tomada de decisão, para o planejamento do cuidado.

O planejamento realizado através do PE caracteriza-se pela tecnologia capaz de orientar a sequência do raciocínio lógico, melhorando dessa forma a qualidade do cuidado por meio da sistematização da avaliação clínica, da identificação dos diagnósticos, da elaboração das intervenções e da monitorização dos resultados do cuidado de Enfermagem (SASSO et al., 2013).

Essa ferramenta garante a realização do cuidado integral e organizado, assim como a continuidade das informações da equipe de enfermagem, permitindo a avaliação da sua eficácia e efetividade, possibilitando modificá-lo de acordo com os resultados na recuperação do paciente (BARRA e SASSO, 2010).

Desse modo, pacientes em regime de hospitalização, em diversas especialidades, podem se beneficiar de uma avaliação clínica de enfermagem que evidencie aspectos importantes relacionados à sua condição clínica. A avaliação clínica efetiva do enfermeiro, certamente auxiliará esse profissional na elaboração dos diagnósticos de enfermagem e do planejamento do cuidado.

A discussão acerca do desenvolvimento, da incorporação e da utilização de tecnologias nos serviços de saúde ganha amplas conotações na atualidade, tanto em nível teórico, principalmente do ponto de vista conceitual, quanto aos impactos da incorporação de tecnologias na prática dos serviços de saúde. E, a enfermagem tem investindo na produção de conhecimentos sobre a temática, envolvendo os múltiplos entendimentos das tecnologias na área da saúde (SILVA; FERREIRA, 2014).

Dentre os diferentes contextos em que a tecnologia pode ser utilizada na enfermagem, a assistencial e a educacional são apontadas como de maior relevância.

As tecnologias em saúde podem ser classificadas em: leve – a composição de relações para implementação do cuidado (por exemplo: vínculo, gestão de serviços,

acolhimento); leve-dura – a construção do conhecimento por meio de saberes sistematizados/estruturados (por exemplo: teorias, modelos de cuidado, cuidado de enfermagem); dura – a utilização de instrumentos, normas e equipamentos tecnológicos (MERHY, 2002).

Por outro lado, a perspectiva da tecnologia como atividade humana e profissional representa uma reflexão importante para a enfermagem, considerando que pode promover a reflexão do processo de trabalho, em especial no que diz respeito às competências profissionais necessárias para a prestação do cuidado (CROZETA et al, 2010).

O uso da tecnologia no cuidado facilita o trabalho do enfermeiro, uma vez que agiliza, traz maior precisão e rapidez nas ações, proporciona maior tempo para a equipe de enfermagem se dedicar ao cuidado, resultando em melhoria da qualidade da assistência (SILVA; FERREIRA, 2014).

Dessa forma, a tecnologia permeia todo o processo de trabalho em saúde, pois colabora na construção do saber, desde o momento da idealização, criação, e implementação do conhecimento, como também, no resultado dessa construção. Portanto, é concomitante processo e produto (SANTOS, 2016).

Diante do exposto, a questão de pesquisa é: "A implantação de uma tecnologia assistencial para avaliação clínica do enfermeiro contribui para o uso do PE no planejamento do cuidado"? Definiu-se como objetivo geral Desenvolver Tecnologias Assistenciais para Avaliação Clínica do Enfermeiro de pacientes em cuidados de saúde no ambiente hospitalar.

Espera-se que o desenvolvimento de uma Tecnologia Assistencial para a avaliação clínica específica do enfermeiro, possa fornecer informações efetivas que tragam elementos para a elaboração de diagnósticos de enfermagem e a definição das melhores estratégias de intervenção, de maneira a consolidar a prática clínica da enfermagem, contribuir para o bom emprego do PE e materializar a SAE.

HIPÓTESES A SEREM TESTADAS

O desenvolvimento de uma Tecnologia Assistencial para a Avaliação Clínica do Enfermeiro contribui para a efetiva utilização do Processo de Enfermagem no

planejamento do cuidado e, conseqüentemente, na consolidação da SAE nas unidades de internação.

OBJETIVOS DA PESQUISA:

– **Objetivo Primário:** Desenvolver Tecnologias Assistenciais para Avaliação Clínica do Enfermeiro de pacientes em cuidados de saúde no ambiente hospitalar.

-**Objetivo Secundários:**

- ✓ Desenvolver Tecnologia Assistencial para Avaliação do paciente em tratamento clínico; (Desenvolvido pela Mestranda Bruna Morelli Bottega)
- ✓ Desenvolver Tecnologia Assistencial para Avaliação do paciente submetido a procedimento cirúrgico; (Desenvolvido pela Mestranda Fabíola Nascimento)
- ✓ Desenvolver Tecnologia Assistencial para Avaliação do paciente crítico; (Desenvolvido pela Mestranda Mariá Comparim)
- ✓ Desenvolver Tecnologia Assistencial para Avaliação do paciente sob cuidados paliativos; (Desenvolvido pela Mestranda Jéssica de Fátima Gomes Pereira)
- ✓ Desenvolver Tecnologia Assistencial para Avaliação do paciente pediátrico em morte encefálica; (Desenvolvido pela Mestranda Vanda Tolari)
- ✓ Desenvolver Tecnologia Assistencial para Avaliação do neonato em cuidados intensivos. (Franciele Rocha)
- ✓ Implantar as tecnologias assistências desenvolvidas nas unidades campo da pesquisa
    - ✓ Desenvolver, validar e implantar protocolos assistenciais de enfermagem;
    - ✓ Desenvolver e implementar tecnologia assistencial para avaliação de enfermagem clínico-cirúrgica;

RELEVÂNCIA SOCIAL

A sistematização da Assistência (SAE) no cenário da saúde proporciona organização no desenvolvimento do cuidado de enfermagem, tornando-se uma estratégia metodológica que promove a liderança do enfermeiro.

O Processo de Enfermagem (PE), considerado um elemento essencial para a materialização da SAE, é uma atividade privativa do enfermeiro, segundo a Resolução 358/2009 do COFEN, considerando que a Enfermagem é constituída por diferentes categorias, incluindo o Enfermeiro, o Técnico de Enfermagem e o Auxiliar de Enfermagem, com diferentes atribuições no processo de cuidar.

O PE se desenvolve em diferentes etapas inter-relacionadas, interdependentes e recorrentes, que inclui a coleta de dados de Enfermagem, a identificação dos Diagnósticos de Enfermagem, o planejamento das intervenções de Enfermagem, a implementação das intervenções e, o monitoramento das respostas dos pacientes aos cuidados prestados.

A primeira etapa se dá por meio do histórico do paciente e do exame físico, dando subsídios para o desenvolvimento das etapas seguintes do Processo de Enfermagem e, requer do enfermeiro competências específicas, exigindo o uso do raciocínio clínico e pensamento crítico. Portanto essa etapa constitui-se na avaliação clínica no paciente sob os cuidados de enfermagem e, tem por finalidade guiar o enfermeiro no estabelecimento de diagnósticos de enfermagem acurados.

Apesar da importância das demais etapas do PE, a avaliação clínica é a base da inter-relação de todas as outras e, por isso, considera-se fundamental o desenvolvimento de tecnologias assistenciais que auxilie o enfermeiro na avaliação clínica diária de pacientes hospitalizados.

Uma tecnologia assistencial para avaliação específica do enfermeiro é uma ferramenta essencial para fornecer informações efetivas que tragam elementos para a elaboração de diagnósticos de enfermagem e a definição das melhores estratégias de intervenção, de maneira a consolidar a prática clínica da enfermagem e contribuir para o bom emprego do PE.

ANTECEDENTES CIENTÍFICOS

A Sistematização da Assistência de Enfermagem (SAE) é um método de organizar, planejar e executar ações sistematizadas, utilizado pelo enfermeiro para assistir o individuo que se encontra sob a assistência de enfermagem (SANTOS, 2014). O Processo de Enfermagem, elemento essencial para a efetivação da SAE, é entendido pelo Conselho Federal de Enfermagem como uma atividade privativa do enfermeiro que subsidia ações de assistência de enfermagem, contribuindo na promoção, prevenção, recuperação e reabilitação da saúde do indivíduo, família e comunidade.

No Brasil, uma das primeiras enfermeiras a estudar com maior rigor o Planejamento do Cuidado foi Wanda de Aguiar Horta, cujas reflexões deram origem a Teoria das Necessidades Humanas Básicas, publicada em 1979. Uma das contribuições do modelo teórico de Horta foi a proposta do processo de enfermagem, até hoje reconhecido e seguido no Brasil (BENEDET et al, 2016).

Wanda Horta (1979) acreditava que para a enfermagem atuar eficientemente, é necessário o desenvolvimento de uma metodologia de trabalho fundamentada no método científico, a qual denominou "Processo de Enfermagem".

Dessa forma, para a construção dessa metodologia de trabalho Wanda Horta se baseou na Teoria das Necessidades Humanas Básicas de Maslow e, na denominação de Necessidades Psicobiológicas, Psicossociais e Psicoespirituais de João Mohana. (ALMEIDA MA; LUCENA AF,2011).

Para Horta (1979), p.35:

> "O processo de enfermagem é a dinâmica das ações sistematizadas e inter-relacionadas, visando a assistência ao ser humano. Caracteriza-se pelo inter-relacionamento e dinamismo de suas fases ou passos".

Inicialmente, em 1974, Wanda Horta apresenta a metodologia ou Processo de Enfermagem em seis fases: histórico, diagnóstico, plano assistencial, plano de cuidados, evolução e prognóstico.

Atualmente o PE inclui cinco diferentes fases para sua efetivação, a saber: a **Coleta de Dados**, que inclui o histórico do paciente e o exame físico; o **Diagnóstico de Enfermagem** a partir do levantamento de dados clínicos e queixas do paciente; o **Planejamento das Intervenções** necessárias para a melhor assistência; a **Implementação das Intervenções**; e a **Avaliação de Enfermagem** que evidencia os efeitos, as repercuções e os benefícios dos cuidados prestados.

Dessa forma, no desenvolvimento do PE o enfermeiro realiza a interpretação e o agrupamento dos dados que auxiliam na tomada de decisão. Portanto, o PE é a base para o planejamento das intervenções de enfermagem fundamentadas pelo conhecimento técnico cientifico da área, respeitando valores e crenças morais (COFEN, 2009; ALMEIDA, 2010; MULLER et al., 2016).

A avaliação diária do paciente pelo enfermeiro deve ser realizada detalhadamente e, para que isso ocorra de forma eficaz sem demandar tempo excessivo da rotina dos enfermeiros, a utilização de uma Tecnologia Assistencial (TA) que aborde os tópicos essenciais, dinamiza esse processo (RÊGO; SANTOS e SANTOS, 2014). Essas pesquisadoras afirmam que a compreensão da tecnologia como um conceito abrangente, bem como discutir e aprimorar essa compreensão possibilita entender o processo de trabalho do enfermeiro e, consequentemente, a sistematização da assistência.

Reconhecidamente, a enfermagem tem sido privilegiada desde 1982, quando iniciou um movimento para a classificação das práticas de cuidado, pela North American Nursing Diagnósis Association (NANDA), com o objetivo de desenvolver uma linguagem específica para os profissionais da enfermagem. Diferente do Código Internacional de Doenças (CID), a NANDA se preocupa em descrever a reação da pessoa e da família, diante de um agravo à saúde, seja por uma doença ou uma situação de risco (BOTOSSO, 2014).

A American Nurses Association (ANA) reconhece três linguagens de enfermagem: os diagnósticos elaborados pela NANDA Internacional (Norht American Nursing Diagnósis Association); as intervenções da Classificação das Intervenções de Enfermagem (NIC) e os resultados da Classificação dos Resultados de Enfermagem (NOC). (JOHNSON et al., 2009)

Além destas, são reconhecidas a CIPE (Classificação Internacional para a Prática de Enfermagem) e o inventário vocabular da CIPESC (Classificação Internacional das Práticas de Enfermagem em Saúde Coletiva) (MALUCELLI A. et. Al, 2010)

CASUÍSTICA

Modelos de avaliação clínica do enfermeiro têm sido experimentados em diversos cenários de cuidados a saúde. Hospitais em geral, incluindo o CHC, campo desta pesquisa, utilizam-se de um modelo biomédico para planejar o cuidado de enfermagem. Mas, considerando as características do processo de trabalho do enfermeiro, ainda que de forma incipiente, já há propostas de modelos de avaliação clínica específica do enfermeiro.

Rêgo, Santos e Santos (2014), considerando a avaliação clínica diária realizada pelos enfermeiros, crucial para manter atualizada a condição de cada paciente e dinamizar a assistência, desenvolveram um estudo com o objetivo de elaborar e obter consenso sobre uma tecnologia assistencial para avaliação clínica de enfermagem no período pós-operatório. A Tecnologia foi validada por um grupo de nove juízes-especialistas e os pesquisadores concluíram que a tecnologia assistencial elaborada auxilia os enfermeiros a executarem de maneira dinâmica e eficaz a assistência de enfermagem. Os pesquisadores alertam, ainda, que a tecnologia assistencial é essencial, desde que seja devidamente elaborada, julgada e consensualizada para utilização.

MÉTODO

Método para a validação e implantação do aplicativo TIS – Cuidados Paliativos

DESENHO DA PESQUISA

Trata-se de pesquisa metodológica, para validação, avaliação e implementação de software desenvolvido com a finalidade de subsidiar a avaliação clínica de enfermeiros em pacientes sob CP. Este tipo de pesquisa costuma envolver modelos de pesquisa com método misto, com a intenção de combinar elementos de ambas as abordagens para aprofundar o entendimento (POLIT; BECK, 2018).

Na enfermagem, a pesquisa metodológica tem sido utilizada em quatro modalidades de estudos: desenvolvimento de instrumentos de medida; desenvolvimento de tecnologias para processos de educação, gerência e de assistência; validação de

diagnósticos, resultados e intervenções de enfermagem; tradução e adaptação transcultural de instrumentos produzidos em outros países (TEIXEIRA, 2019).

ASPECTOS ÉTICOS

Esta pesquisa é vinculada ao Projeto Temático intitulado "Tecnologias para Qualificar e Consolidar a Sistematização da Assistência de Enfermagem nos Diferentes Cenários da Prática Profissional", aprovado pelo Comitê de Ética em Pesquisa (CEP) do Complexo Hospital de Clínicas da Universidade Federal do Paraná (CHC-UFPR), conforme parecer de nº 2.947.877 (Anexo 1), como prevê a Resolução nº466, de 12 de CHC-UFPR dezembro de 2012, que trata de diretrizes e normas regulamentadoras de pesquisas envolvendo seres humanos (BRASIL, 2012).

Durante o desenvolvimento da pesquisa, seguir-se-ão os princípios de autonomia, beneficência, não maleficência e justiça. Garantir-se-ão a confidencialidade, o anonimato dos participantes, o sigilo das informações, bem como o uso dos resultados, unicamente para fins de publicação em revistas científicas (BRASIL, 2012).

LOCAL DE PESQUISA

O local de desenvolvimento da pesquisa é o Programa de Pós-graduação em Enfermagem, tendo como cenários para implantação, inicialmente, o CHC-UFPR e, posteriormente, outros ambientes de cuidados paliativos.

PARTICIPANTES

Dois grupos de participantes serão selecionados para esta pesquisa. O primeiro grupo será composto por enfermeiros especialistas em cuidados paliativos, para validação do conteúdo do aplicativo.

O segundo grupo será composto por profissionais da área de Tecnologia de Informação (TI) e enfermeiros que assistem pacientes em cuidados paliativos, os quais participarão como juízes para avaliação de qualidade do produto.

PROTOCOLO DE PESQUISA

O aplicativo móvel denominado AVALIA TIS – Cuidados Paliativos, pela complexidade, foi desenvolvido com o apoio técnico das empresas Associação Júnior de Desenho Industrial e Associação Júnior de Consultoria em Informática da UFPR.

Para validar, avaliar e implementar a referida tecnologia, esta pesquisa terá o desenvolvimento em diferentes etapas e fases, como mostra a Figura 18.

Figura 1 - Etapas e Fases para o desenvolvimento da pesquisa

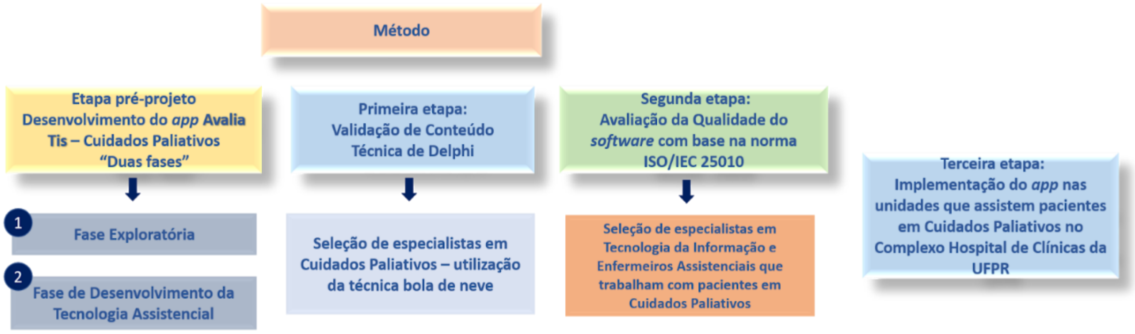

FONTE: A autora (2021).

Etapas da Pesquisa

Pré-projeto

Esta etapa incluiu duas fases já realizadas para o desenvolvimento do aplicativo:

A **primeira fase** – exploratória – incluiu revisão de literatura nas bases de dados científicos em relação à avaliação clínica e busca de informações junto aos participantes, neste caso, enfermeiros da prática clínica, quanto aos aspectos que consideram na avaliação de pacientes em cuidados paliativos.

A **segunda fase** – desenvolvimento da tecnologia – incluiu a definição dos requisitos e mapa conceitual do aplicativo e geração das alternativas de implementação e prototipagem.

Primeira Fase – Validação de Conteúdo

Esta etapa tem como objetivo validar o conteúdo que compõe o aplicativo AVALIA TIS – Cuidados Paliativos, por um comitê de expert no tema. Para o desenvolvimento, utilizar-se-á a Técnica Delphi, que permite a avaliação de informações, para alcançar concordância sobre determinado tema, ocorre por meio de validações articuladas em fases e etapas (CASTRO; REZENDE, 2009).

A operacionalização da técnica inicia com a seleção e o contato dos especialistas. Posteriormente, a elaboração de um questionário para aplicar aos participantes. Caso, na primeira rodada da avaliação, não haja consenso dos juízes em relação ao conteúdo e/ou à presença de recomendações para ajuste, uma segunda rodada é realizada. Após o atendimento às recomendações da primeira rodada, um novo questionário deve ser encaminhado para segunda rodada. Assim ocorre sucessivamente, até atingir nível de consenso (WRIGHT; GIOVINAZZO, 2000).

Para esta pesquisa, propõem-se duas rodadas de avaliação pelo grupo de juízes, pois o processo de desenvolvimento do software já envolveu uma etapa que buscou sugestões dos profissionais que assistem pacientes em cuidados paliativos, acerca dos aspectos importantes na avaliação clínica de enfermeiros. Desta forma, as etapas se estabelecerão seguindo Marques e Freitas (2018):

a) **Seleção dos especialistas** - para composição do comitê de avaliação, a pesquisadora selecionará amostragem não probabilística intencional, utilizando-se da técnica Bola de Neve, na qual um especialista indica o outro para compor o grupo de juízes. Os critérios de inclusão para seleção dos juízes serão baseados no sistema de pontuação de Fehring (1987) para estudos de validação, descritos na Tabela 1.

Tabela 1 – Critérios de inclusão – *expertise*

Critérios de inclusão	Pontuação
Experiência na área de ensino ou assistência em Cuidados Paliativos;	3
Mestre em enfermagem com dissertação na área de Cuidados Paliativos e/ou Processo de Terminalidade e/ou Especialista nas referidas áreas;	2
Doutor em enfermagem com a tese em Cuidados Paliativos e/ou Processo de Terminalidade;	3
Pesquisas publicadas com ênfase no tema Cuidados Paliativos e/ou Processo de Terminalidade;	2
Participar de laboratórios de pesquisa na área de enfermagem em Cuidados Paliativos e/ou Processo de Terminalidade.	1

FONTE: Adaptado de Fehring (1987).

Para a amostra, os juízes deverão apresentar escores maiores ou iguais a cinco pontos (FEHRING, 1987). Excluir-se-ão os participantes que não responderem ao questionário em até dez dias após recebimento.

b. **Construção do questionário 1** – elaborado com tópicos relacionados às interfaces do aplicativo móvel, a saber: histórico de enfermagem; escalas utilizadas na avaliação do paciente sob CP; necessidades psicobiológicas, psicossociais e psicoespirituais; avaliação da família. As respostas serão apresentadas em escala tipo *Likert*, que servirá para avaliar a adequação do conteúdo: (1) não adequado; (2) pouco adequado; (3) adequado; (4) muito adequado.

c. **Primeiro contato com os especialistas, convite para participação na pesquisa e envio do questionário 1** - inicialmente, a pesquisadora enviará e-mail para o possível participante do grupo de juízes, no qual apresentará a pesquisa e solicitará a participação. Aqueles que aceitarem receberão o Termo de Consentimento Livre e Esclarecido (TCLE) (APÊNDICE 1), uma carta de orientação da Técnica Delphi (APÊNDICE 2), o questionário da primeira rodada (APÊNDICE 3), construído no *Google Forms*, e o link para download do aplicativo. Este questionário também contará com um campo para que os juízes

possam apresentar recomendações. Solicitar-se-á, ainda, a indicação de outro especialista para compor o grupo de juízes.

d. **Recebimento das respostas ao questionário 1 e análise das respostas -** a concordância entre as respostas dos juízes será obtida pelo Índice de Validade de Conteúdo (IVC) que avaliará cada item do instrumento e, após, realizar-se-á a avaliação em totalidade. O IVC é um método bem utilizado na área da saúde e aponta que o foco principal da validação de conteúdo é determinar se os itens elencados apresentam adequação do conteúdo proposto. Para o cálculo, a recomendação é de seis ou mais juízes e taxa de concordância não inferior a 0,78 (ALEXANDRE; COLUCI, 2011).
e. **Construção e envio do questionário 2 com feedback -** se após análise das respostas do questionário 1, houver necessidade de ajuste no conteúdo, realizar-se-ão as alterações sugeridas e, posteriormente, a pesquisadora enviará novo link com aplicativo e novo questionário aos juízes.
f. **Recebimento das respostas ao questionário 2 e análise das respostas -** a concordância entre as respostas dos juízes será obtida pelo Índice de Validade de Conteúdo (IVC) que avaliará cada item do instrumento e, após, realizar-se-á a avaliação em totalidade (ALEXANDRE; COLUCI, 2011).
g. **Final do processo e escrita do relatório final** - pretende-se estabelecer duas rodadas, para que haja a validação do conteúdo proposto, contudo, entende-se que o processo de rodadas de questionários termina quando existir consenso entre as respostas obtidas (OSBORNE *et al.*, 2003). Desta maneira, caso haja divergências consideráveis ao final da segunda rodada, a pesquisadora poderá realizar novas rodadas até alcançar estabilidade nas respostas obtidas e finalização desta etapa da pesquisa, conforme mostra a figura a seguir.

Figura 2- Rodadas de questionários técnica Delphi

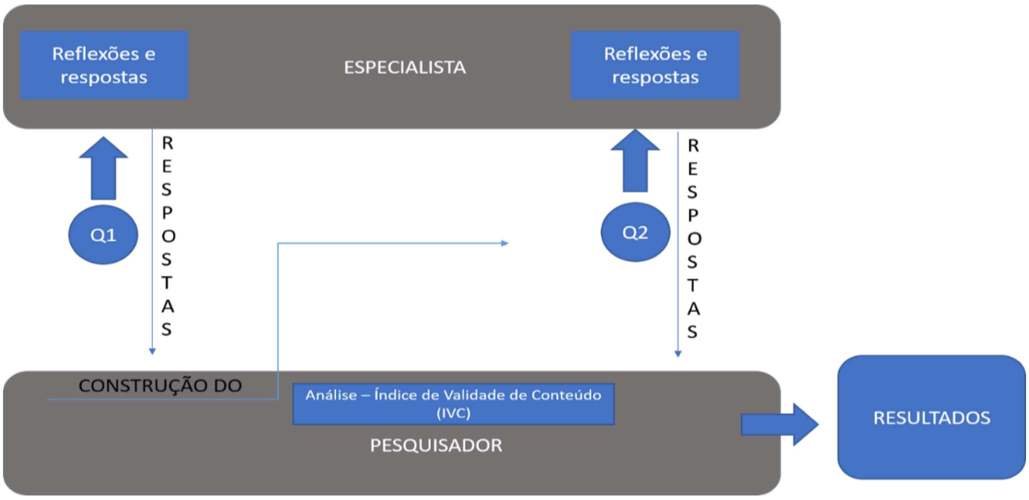

FIGURA: Adaptado de Marques e Freitas (2018).

Segunda Etapa – Avaliação de Qualidade do Software

Esta etapa tem como objetivo avaliar a qualidade do aplicativo desenvolvido. De acordo com as recomendações da ISO/IEC 8402, a qualidade de um software é definida por um conjunto de características que oferecem nível de satisfação, para que o produto desenvolvido supra as necessidades de quem os utiliza (ISO/IEC 8402, 1994).

No intuito de avaliar a qualidade do software desenvolvido, a pesquisadora utilizará o Modelo de Qualidade de Produto da norma ISO/IEC 25010 e o Processo de Avaliação da norma ISO/IEC 25040.

Assim, o processo de avaliação da qualidade seguirá as seguintes fases, conforme ISO/IEC 25040:

a) Estabelecer Requisitos da Avaliação

Definir-se-ão os objetivos e os critérios da avaliação. Esta fase utilizará as características de qualidade da norma ISO 25010 para Avaliação da Qualidade do Produto, a saber:

- Adequação funcional;
- Eficiência de desempenho;
- Compatibilidade;
- Usabilidade;
- Confiabilidade;
- Segurança;
- Manutenibilidade;

- Portabilidade.

b) Especificar a avaliação, definindo as métricas de qualidade, os níveis de pontuação e critérios para julgamento

- Para o processo de avaliação de qualidade, utilizar-se-á um formulário adaptado de Santos (2019), desenvolvido com base nos formulários de Sperandio (2008) e Oliveira e Peres (2015), para avaliar a qualidade de um aplicativo de apoio à decisão de enfermeiros na prevenção de lesões de pele em recém-nascidos internados. Os formulários de Sperandio (2008) e Oliveira e Peres (2015) são validados quanto à clareza, compreensão e objetividade, seguindo os níveis de pontuação da ABNT NBR ISO/IEC 14598-6.
- Os formulários a serem aplicados terão níveis de pontuação, com base na ABNT NBR ISO/IEC 14598-6 - (C) Concordo; (D) Discordo; (NA) Não se Aplica; e, Comentários. O nível (C) significa que o item atende ao requisito de qualidade; o nível (D) que o item não atende ao requisito de qualidade; e o nível NA (Não se Aplica) referente ao item não se aplica ou não foi avaliado.
- Serão considerados adequados os itens que obtiverem o percentual de concordância maior do que 70%, conforme a escala de avaliação para subcaracterísticas adaptada de Sperandio (2008), a partir da ABNT NBR ISO/IEC 14598-6.

c) Projetar, Planejar as Atividades de Avaliação

Trata-se de quando e como a avaliação será realizada. Definição do cronograma das atividades e recursos necessários: humanos, hardware, software e orçamento previsto.

- Selecionar-se-ão dois grupos de juízes, constituídos por enfermeiros que assistem pacientes em cuidados paliativos e profissionais da área de Tecnologia da Informação (TI). Para a avaliação, utilizar-se-á das recomendações da ABNT/ISO/IEC 25062 (2011) que indica amostragem mínima de oito avaliadores para cada categoria de usuários.

A seleção dos profissionais para compor o grupo de juízes se dará por meio da técnica Bola de Neve, na qual um especialista indica outro, até obter a composição do grupo pretendido. Os critérios de inclusão para seleção dos grupos de participantes desta

etapa serão baseados no sistema de pontuação de Fehring (1987) para estudos de validação, descritos nas Tabelas 2 e 3.

Tabela 2 - Critérios de inclusão para seleção dos especialistas em TI.

Critérios de inclusão	Pontuação
Experiência de ensino na área de desenvolvimento de *software*	1
Experiência no desenvolvimento de *software* há pelo menos dois anos	3
Mestre em ciências da computação, com dissertação em desenvolvimento de *software*	2
Doutor em ciências da computação com a tese sobre desenvolvimento de *software*	3
Pesquisas publicadas sobre desenvolvimento de *software*	2

FONTE: Adaptado de Fehring (1987).

Tabela 3 - Critérios de inclusão para seleção dos enfermeiros assistenciais

Critérios de inclusão	Pontuação
Experiência na área de ensino ou assistência em Cuidados Paliativos;	3
Mestre em enfermagem com dissertação na área de Cuidados Paliativos e/ou Processo de Terminalidade e/ou Especialista nas referidas áreas;	2
Doutor em enfermagem com a tese em Cuidados Paliativos e/ou Processo de Terminalidade;	3
Pesquisas publicadas com ênfase no tema Cuidados Paliativos e/ou Processo de Terminalidade;	2
Participar de laboratórios de pesquisa na área de enfermagem em Cuidados Paliativos e/ou Processo de Terminalidade.	1

FONTE: Adaptado de Fehring (1987).

d) Executar a avaliação, realizando medidas, aplicando critérios de decisão para medidas de qualidade e avaliação

- A avaliação será realizada a partir de dois formulários (APÊNDICE 6 e7) do *Google Forms*, enviados para o grupo de enfermeiros e de especialistas em TI, contendo características e questões específicas para as subcaracterísticas de qualidade.

  Os participantes serão convidados, por meio de carta-convite (APÊNDICE 5) enviada por correio eletrônico. Àqueles que concordarem em participar no estudo, serão solicitadas leitura e assinatura do Termo de Consentimento Livre e Esclarecido (TCLE) (APÊNDICE 4).
- As técnicas de medição serão aplicadas em relação aos requisitos de qualidade. Neste momento, os critérios de decisão serão analisados e os resultados registrados.

e) Concluir a avaliação

- Com a finalização das fases anteriores, os dados serão analisados pela pesquisadora. A atividade servirá como entrada para identificação de possíveis pontos de melhoria tanto para o software como para os métodos de avaliação utilizados.

Terceira Etapa - Implementação

Na terceira etapa da pesquisa, o aplicativo já validado será implementado em um hospital de ensino, especificamente, nas unidades de internação que, no período de implementação, tenham leitos para pacientes em CP.

O Complexo Hospitalar lócus do estudo está classificado como a maior instituição hospitalar do Paraná e o quinto maior dentre os hospitais universitários do Brasil. É referência em inúmeras especialidades e compõe 261 consultórios, com total de 498 leitos ativos, distribuídos em 59 especialidades.

Esta etapa da pesquisa será instrumentalizada em duas fases.

a) Primeira fase: **capacitação dos profissionais**

Nesta fase, realizar-se-ão três encontros, com duração de 30 minutos durante o turno de trabalho dos enfermeiros (manhã, tarde e noite). As capacitações seguirão a seguinte ordem:

Avaliação clínica do enfermeiro

O conteúdo do primeiro contato com os enfermeiros assistências abordará a importância da avaliação clínica diária. Para tanto, será utilizada tecnologia do tipo vídeo, em animação 2D, que aborda os aspectos relevantes a serem avaliados na primeira manobra propedêutica do exame físico – inspeção. Ressalta-se que esta estratégia já foi realizada antes da aplicação do questionário para coleta de dados que subsidiou o desenvolvimento do aplicativo móvel.

Avaliação do paciente em cuidados paliativos

Neste encontro, elencar-se-ão os aspectos multidimensionais que devem ser considerados durante a avaliação clínica do paciente em cuidados paliativos. É importante que contemple elementos que compreendam quem é o paciente, preferências, necessidades, dificuldades e expectativas deste, de modo a permitir conhecer a evolução da doença, os tratamentos realizados, as medicações propostas, as decisões clínicas, a evolução e o prognóstico.

AVALIA TIS - Cuidados Paliativos

Por fim, a pesquisadora apresentará o aplicativo AVALIA TIS - Cuidados Paliativos, o objetivo do desenvolvimento da tecnologia e como pode beneficiar o trabalho do enfermeiro durante a realização da avaliação clínica.

b. Segunda fase: **Operacionalização do aplicativo**

Nesta fase, a pesquisadora irá realizar o treinamento dos enfermeiros utilizando estudos de caso referente a temática. Será disponibilizado um tablet, bem como, o e-mail e *WhatsApp* da pesquisadora, para que o participante possa esclarecer dúvidas referentes ao manuseio da tecnologia.

Ressalta-se que esta tecnologia foi aprovada para ser implementada no Complexo Hospital de Clínicas da UFPR, tendo o apoio da Comissão de Sistematização da Enfermagem (COMISAE) para execução nos ambientes onde os CP são realizados.

Método para implantação do App AVALIA TIS – Paciente Clínico

LOCAL DE PESQUISA

O local de desenvolvimento da pesquisa é o Programa de Pós-graduação em Enfermagem, tendo como cenário para implantação o Hospital de Guarnição de Porto Velho.

PARTICIPANTES

Participantes serão enfermeiros que assistem pacientes clínicos no Hospital de Guarnição de Porto Velho.

PROTOCOLO DE PESQUISA

A pesquisa seguirá as fases apresentadas a seguir:

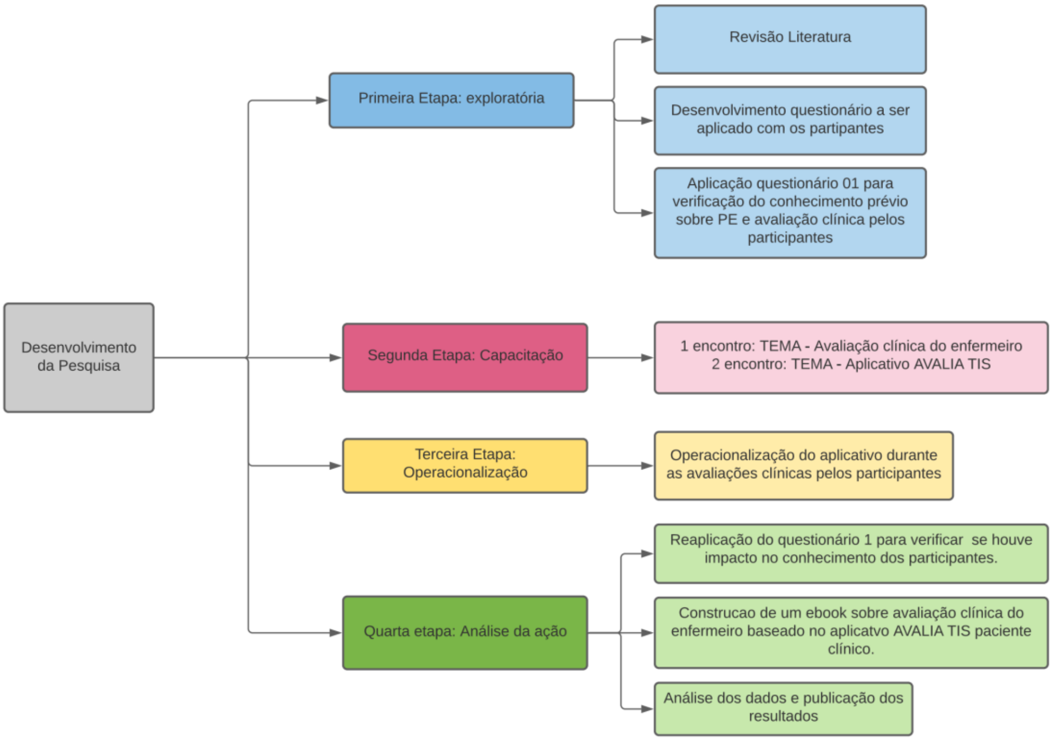

Figura 6. Etapas da Pesquisa Fonte: Autores

Primeira etapa- denominada fase exploratória é constituída por três fases:

Primeira fase: Revisão da Literatura.

Realizada a busca nas bases de dados nacionais e internacionais com a utilização da estratégia de busca apresentada abaixo:

Figura 7. Estratégia de busca da pesquisa

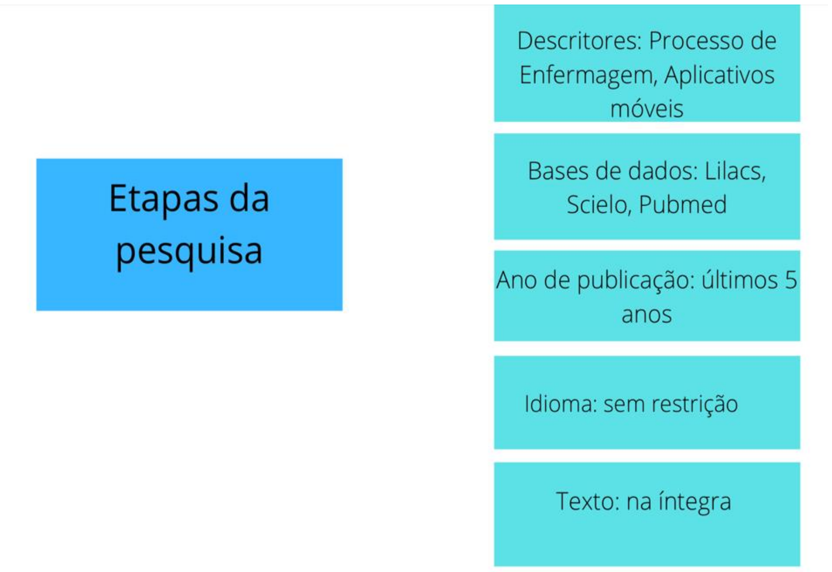

A partir da estratégia de busca adotada, os resultados foram:11 publicações na base de dados Scientific Electronic Library Online (SciELO), 5 na Literatura Latino-Americana e do Caribe em Ciências da Saúde (Lilacs), não foram encontrados artigos com estes descritores associados na base Pubmed.

Considerando as publicações da base SciELO foram encontrados: 1 publicação que descreve a tradução de aplicativo educativo para lavagem das mãos, 6 publicações sobre aplicativos que orientam cuidado/autocuidado lesões de pele, 1 artigo aplicativo sobre orientações em sala de vacina, 1 aplicativo sobre educação de enfermeiros em saúde da família, 1 aplicativo sobre PE no atendimento pré hospitalar e 1 aplicativo sobre histórico e diagnóstico de enfermagem.

Dentre as publicações da base de dados Lilacs, 4 dizem respeito a dissertações de mestrado de enfermagem da Universidade Federal do Paraná, evidenciando a escassez de produções que associam a tecnologia por meio de aplicativos ao PE. A outra publicação aborda 1 aplicativo sobre o processo de enfermagem aplicado a pacientes de unidades neonatais.

Após leitura dos títulos e resumos, foram selecionados os 5 artigos que relacionaram o processo de enfermagem, e as 4 dissertações com a mesma temática.

Segunda fase: Elaboração do questionário.

Baseado na revisão da literatura foi elaborado um questionário semi-estruturado (apêndice A) para identificação dos conhecimentos prévios dos participantes a respeito da avaliação clínica do enfermeiro e do Processo de Enfermagem. Este foi construído na plataforma Google forms.

4.5.1.3 Terceira fase: Recrutamento dos participantes e Aplicação do questionário 1

Para o recrutamento dos participantes, a pesquisadora enviará uma carta convite contendo informações a respeito da pesquisa via e-mail. Àqueles que aceitaram participar da pesquisa, será enviado o Termo de Consentimento Livre e Esclarecido (Apêndice C) e o questionário 1 (Apêndice A).

As respostas obtidas a partir da aplicação do questionário 1 subsidiarão os temas a serem abordados nos encontros de capacitação a fim de mitigar as dúvidas mais frequentes e possibilitar a compreensão dos participantes sobre a importância da implementação do PE na prática clínica.

Segunda Etapa: Capacitação

Nesta fase serão realizados dois encontros com duração de 30 minutos durante o turno de trabalho dos enfermeiros (manhã, tarde e noite) onde a pesquisadora irá desenvolver os temas por meio de uma aula teórica.

Os encontros seguirão a seguinte ordem:

1) Primeiro encontro: Avaliação clínica do enfermeiro

   O conteúdo deste primeiro contato com enfermeiros assistenciais abordará a importância da avaliação clínica diária. Para tanto, além da explanação teórica, será utilizado uma tecnologia educacional, do tipo vídeo em animação 2D, desenvolvido por um grupo de mestrandos do Programa de Pós-Graduação em Enfermagem Profissional, que apresenta os aspectos clínicos possíveis de ser avaliados na primeira etapa do exame físico – a inspeção (PONTES, *et.al,* 2019).

O material possui o Certificado de Produto Brasileiro, sob o nº **B19-000629-00000**, pela Agência Nacional do Cinema (ANCINE), e foi publicado em um canal do YouTube, com acesso livre. As cenas da versão final do vídeo estão apresentadas no Anexo 2.

2) Segundo encontro: AVALIA TIS - Paciente Clínico

   No segundo encontro, a pesquisadora irá apresentar o aplicativo avalia TIS- Paciente Clínico, mostrando o objetivo do desenvolvimento da tecnologia e suas contribuições para o PE durante a avaliação clínica.

Terceira etapa: Operacionalização do aplicativo AVALIA TIS – Paciente Clínico.

Nesta etapa, os participantes serão cadastrados e utilizarão o aplicativo AVALIA TIS – Paciente Clínico para realização das avaliações clínicas diárias dos pacientes internados no setor de clínica médica. Será disponibilizado um tablet com acesso a internet 4G para possibilitar a utilização do aplicativo.

Para preservar a privacidade dos pacientes e a não exposição dos dados pessoais, serão incluídos no aplicativo códigos previamente estabelecidos para sua identificação. Os dados pessoais serão incluídos manualmente na evolução e anexados aos prontuários.

O objetivo da utilização do aplicativo será informado aos pacientes e estes poderão autorizar ou não a utilização de seus dados de saúde. Caso autorizem, será disponibilizado o termo de consentimento (Apêndice D), para atender a Lei Geral de Proteção dos Dados n.13.709 de 14 de agosto de 2018 (BRASIL, 2018).

Quarta etapa: Análise da ação.

Esta etapa será constituída por três fases: reaplicação do questionário 1, construção de um *ebook* sobre avaliação clínica do enfermeiro e análise dos dados obtidos.

Primeira fase:

Será reenviado o questionário 1 aos participantes com objetivo de verificar se a utilização do aplicativo gerou impacto no conhecimento a respeito da avaliação clínica do enfermeiro e do PE. As informações obtidas serão tabuladas no editor Microsoft Excel, 365 personal (quantitativos) e Microsoft Word 365 personal (qualitativos) e comparadas com as respostas dadas anteriormente.

Segunda fase:

Será desenvolvido um *ebook*, baseado no aplicativo AVALIA TIS - Paciente Clínico, com objetivo de instruir enfermeiros sobre a primeira etapa do PE: avaliação clínica. Este material poderá ser usado em situações de ensino acadêmicas, educação continuada ou permanente, e ainda, nas fases educativas que antecedem a operacionalização do aplicativo para as instituições que incorporarem essa tecnologia nas práticas profissionais.

A criação do e-book seguirá as seguintes etapas:

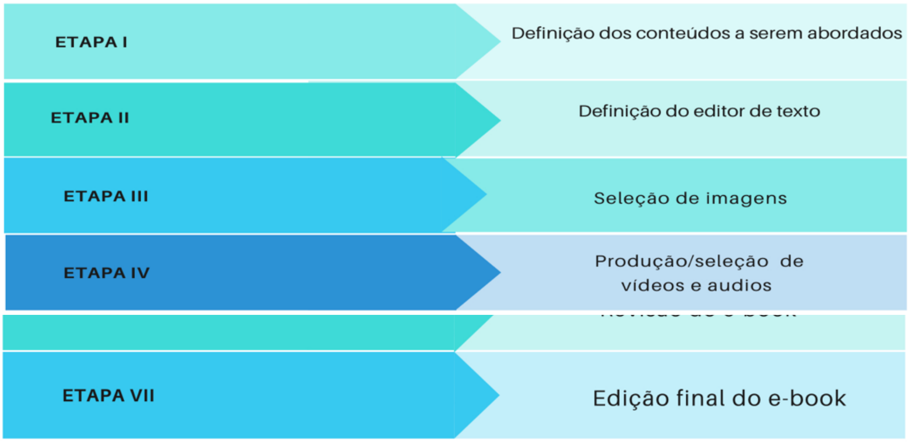

Etapa I- Definição dos conteúdos a serem abordados

Após a revisão da literatura e análise da intervenção proposta serão levantados os conteúdos a serem incluídos no ebook sobre a a avaliação clinica do enfermeiro (anamnese+ exame físico).

Etapa II- Definição do editor de texto

A definição do editor de texto se dará a partir da análise de profissionais de designer e informática contratados para edição do instrumento proposto, pela necessidade de outras áreas do conhecimento.

Etapa III- Seleção de imagens

A seleção de imagens será feita preferencialmente em bancos de imagens gratuitas, a partir da observação das licenças para utilização e créditos para ao autor da imagem, se for o caso.

Etapa IV- Produção/Seleção de vídeos e áudios

Serão produzidos/selecionados vídeos relacionados à temática para facilitar a aprendizagem das etapas da exame físico do enfermeiro a serem inseridos no e-book.

O ebook terá a opção de ser narrado para os usuários que assim desejarem. A opção de audiobook facilitará o acesso ao conteúdo.

Etapa V- Editoração do e-book

A editoração será feita por uma empresa contratada para este fim.

Etapa VI- Revisão do e-book

A revisão do e-book será realizada pelas pesquisadoras envolvidas no projeto de pesquisa.

Etapa VII- Edição final do e-book

Após aprovação da equipe de pesquisadores, o e-book será finalizado pela empresa contratada e estará pronto para registro e divulgação.

Na terceira fase será realizada a análise dos dados e a publicação dos resultados para a instituição e para a comunidade acadêmica.

MÉTODO

Trata-se de uma pesquisa exploratória, a ser realizada no Complexo Hospital de Clínicas e Hospital Infantil Waldemar Monastier, localizados na cidade de Curitiba e Campo Largo – Paraná, respectivamente.

As pesquisas exploratórias são desenvolvidas com o intuito de proporcionar visão geral acerca de determinada situação. Também pode ser realizada quando o tema pesquisado é pouco explorado e a formulação de hipóteses precisas e operacionáveis tornam-se difícil (GIL, 2008).

Para Gil (2008), p. 27:

> "As pesquisas exploratórias têm como principal finalidade desenvolver, esclarecer e modificar conceitos e ideias, tendo em vista a formulação de problemas mais precisos ou hipóteses pesquisáveis para estudos posteriores".

DESENVOLVIMENTO DA PESQUISA

Esta pesquisa se desenvolverá em duas fases, descritas a seguir:

PRIMEIRA FASE DA PESQUISA

Nessa fase se dará a construção da Tecnologia Assistencial para a Avaliação Clínica do enfermeiro.

Coleta de dados

Os dados serão obtidos em duas etapas, no período de agosto de 2018, logo após a aprovação do Comitê de Ética em Pesquisa, a fevereiro de 2019.

Primeira etapa da coleta de dados: **a)** busca bibliográfica, em bases de dados científicos, com o objetivo de aprofundamento teórico do objeto de pesquisa; **b)** busca

de informações junto aos participantes da pesquisa, em relação a sua prática na avaliação clínica do paciente sob seus cuidados.

Nessa fase, a obtenção dos dados junto aos participantes, será realizada individualmente por um dos pesquisadores, que aplicarão um instrumento que contempla três itens: dados sócios demográficos; formação acadêmica e experiência profissional; conhecimento e prática profissional da avaliação clínica. A coleta será realizada em horário previamente agendado de acordo com a disponibilidade do participante e, ocorrerá na sua unidade de trabalho.

Após a obtenção dos dados dessa 1ª etapa, a partir da revisão de literatura e das informações colhidas dos enfermeiros participantes na pesquisa, se iniciará a construção das Tecnologias Assistenciais propostas.

Análise de dados (1ª etapa):

A análise dos dados será realizada a partir da triangulação dos dados obtidos – informações dos participantes da sua prática clínica – dados da literatura –.

Segunda etapa da coleta de dados: nessa etapa os dados serão obtidos por meio de dois encontros entre pesquisador e participantes. Os encontros serão agendados em consenso. Ocorrerão em uma sala reservada previamente nos espaços do Complexo Hospital de Clínicas e, terão espaço para:

- Discussão sobre "Avaliação Clínica do Enfermeiro para a efetivação do Processo de Enfermagem";
- Apresentação da Tecnologia Assistencial para Avaliação Clínica do Enfermeiro aos participantes.

Após apreciação da tecnologia, pelos participantes, será solicitada uma avaliação e possíveis contribuições para o aprimoramento da tecnologia, considerando a *expertise* desses enfermeiros. Esses dados serão registrados em um diário de campo, para posterior avaliação e ajustes necessários na Tecnologia desenvolvida.

<u>SEGUNDA FASE DA PESQUISA</u>

Na segunda fase ocorrerá a implantação da Tecnologia Assistencial desenvolvida, nas unidades campos desta pesquisa. Com o objetivo de avaliar a usabilidade da Tecnologia, esta será disponibilizada para utilização na avaliação clínica dos pacientes sob os cuidados dos enfermeiros assistenciais por um período de 60 dias consecutivos.

Durante esse período será disponibilizado aos enfermeiros um instrumento para o registro das avaliações realizadas, utilizando-se da Tecnologia proposta.

Após, realizar-se-á uma análise dos dados registrados, com o objetivo de identificar se a implantação da Tecnologia Assistencial para Avaliação Clínica do Enfermeiro desenvolvida contribui para o uso do PE no planejamento do cuidado.

Aspectos éticos:

Para o desenvolvimento desta pesquisa, inicialmente o Projeto de Pesquisa foi submetido a apreciação do Programa de Pós-Graduação em Enfermagem Profissional da UFPR e, após, solicitado a concordância junto as Chefias das Unidades envolvidas para o seu desenvolvimento. Após sua aprovação, realizado o encaminhamento do projeto ao Comitê de Ética em Pesquisa (CEPE) do Complexo Hospital de Clínicas da Universidade Federal do Paraná, conforme prevê a Resolução nº466, de 12 de dezembro de 2012, que trata de diretrizes e normas regulamentadoras de pesquisas envolvendo seres humanos.

Durante todo o desenvolvimento do estudo serão seguidos todos os aspectos éticos previstos na Resolução nº 466/2012 do Conselho Nacional de Saúde (CNS). Esta resolução possui os quatro princípios básicos da bioética (autonomia, beneficência, não maleficência e justiça) e, visa assegurar os direitos e deveres da comunidade científica, dos sujeitos da pesquisa e do Estado e no que se refere à confidencialidade, anonimato dos participantes, sigilo das informações e uso dos resultados, unicamente para fins de publicação em revistas científicas.

PARTICIPANTES

A seleção dos participantes ocorrerá de forma intencional, em função da relevância que eles apresentam em relação ao tema.

Serão participantes desta pesquisa todos os enfermeiros assistenciais que atuam nos três turnos de trabalho das Unidades de: Terapia Urgência e Emergência Adulto; Unidade de Terapia Intensiva Pediátrica; Unidade de Clínica Médica e Unidade de Cirúrgica do complexo Hospital de Clínicas da UFPR e, da Unidade de Terapia Intensiva Neonatal do Hospital Infantil Waldemar Monastier.

RESULTADOS DO ESTUDO

Os pesquisadores têm o comprometimento de divulgar os resultados da pesquisa nas instituições envolvidas, para que os participantes possam conhecer sua contribuição no cumprimento dos objetivos propostos. Assim como em eventos e periódicos científicos.

PLANOS PARA O RECRUTAMENTO DO PARTICIPANTE DA PESQUISA

Os participantes serão convidados individualmente e pessoalmente, por um dos pesquisadores, na unidade da sua prática profissional. Será apresentado aos participantes, o objetivo da pesquisa, assim como os procedimentos a serem realizados para a obtenção dos dados e, a forma de sua participação.

Serão informados, ainda, quanto a garantia do anonimato e do direito de ser esclarecido sobre qualquer dúvida, durante o desenvolvimento da pesquisa e, que poderá desistir de participar em qualquer momento sem prejuízo nas suas atividades profissionais.

Participarão da pesquisa os enfermeiros em atividade profissional nas unidades, campo desta pesquisa, que aceitarem participar e assinarem o termo de consentimento livre e esclarecido. Serão excluídos da amostra os enfermeiros que não atenderem os critérios de inclusão.

C**RITÉRIOS DE INCLUSÃO**

- ✓ Atuar como enfermeiro assistencial nas Unidades campo da pesquisa;
- ✓ Interesse em participar da discussão do tema da pesquisa;

- ✓ Concordar em participar do estudo e assinar o Termo de Consentimento Livre e Esclarecido (TCLE) (APÊNDICE 01).

Como **critérios de exclusão**:

- ✓ Estar afastado das atividades assistenciais no período de coleta de dados;
- ✓ Declarar não haver interesse em participar da discussão do tema da pesquisa.

ANÁLISE DE MÉRITO, DE BENEFÍCIOS E RISCOS

Mérito: A pesquisa propõe o desenvolvimento de uma tecnologia assistencial que auxilie o profissional enfermeiro na avaliação clínica do paciente, com o objetivo de obter dados que permitam a identificação de problemas ou necessidades do paciente, assim como a elaboração de um plano de cuidados.

A possibilidade de o enfermeiro avaliar aspectos importantes que subsidiem a tomada de decisão, utilizando-se do raciocínio clínico na elaboração de intervenções para o cuidado, poderá promover maior adesão desses profissionais na utilização do Processo de Enfermagem na sua prática clínica.

O método é adequado, considerando os objetivos propostos, os quais exigem conhecimento aprofundado do objeto de estudo, neste caso, avaliação clínica do enfermeiro. A contribuição dos participantes na construção da tecnologia assistencial a partir da sua *expertise*, certamente, aumentará as chances de adesão ao uso da tecnologia e, consequentemente, na efetivação do Processo de Enfermagem para o planejamento do cuidado.

Benefícios: O desenvolvimento de uma Tecnologia Assistencial, para avaliação clínica específica do enfermeiro, auxiliará esse profissional na elaboração dos diagnósticos de enfermagem e do planejamento do cuidado. Os pacientes hospitalizados se beneficiam por meio da avaliação clínica do enfermeiro, que evidencie aspectos importantes relacionados à sua condição clínica.

Riscos: Há risco de constrangimento dos participantes na fase de obtenção dos dados, por meio de consulta da sua prática em relação a avaliação clínica, utilizando-se da sua *expertise*.

MEDIDAS DE PROTEÇÃO OU MINIMIZAÇÃO DE QUALQUER RISCO EVENTUAL

Os pesquisadores terão o compromisso de agir de forma neutra em relação as informações que os participantes disponibilizarem sobre sua prática clínica, para protege-los do risco de constrangimento ou minimizá-lo.

Caso ocorra constrangimento por parte de algum participante da pesquisa, o grupo de pesquisadores tratará individualmente, garantindo o anonimato dos profissionais nos relatos realizados aos pesquisadores, assim como oferecerá a liberdade de afastamento do participante na pesquisa.

DESFECHO PRIMÁRIO

Desenvolvimento de uma Tecnologia Assistencial para Avaliação Clínica Sspecífica do Enfermeiro.

DURAÇÃO TOTAL DA PESQUISA

A pesquisa proposta será desenvolvida a partir da aprovação pelo Sistema CEP-CONEP. Os pesquisadores se comprometem de que a pesquisa terá início somente após aprovação do CEP/HC com sua finalização prevista para outubro de 2021, totalizando 24 meses. As atividades a serem desenvolvidas estão descritas conforme Cronograma a seguir:

	**2021**			**2022**												**2023**										
ATIVIDADE	Out	Nov	Dez	Jan	Fev	Mar	Abr	Mai	Jun	Jul	Ago	Set	Out	Nov	Dez	Jan	Fev	Mar	Abr	Mai	Jun	Jul	Ago	Set	Out	Nov
Encaminhamento Emenda ao CEP																										
Qualificação Projeto de Pesquisa																										
Etapa II Avaliação de qualidade do produto app Cuidados Paliativos																										
Análise dos dados ajustes no app Cuidados Paliativos																										
Ajustes no app, conforme orientação dos juízes																										
Elaboração do artigo (referente aos resultados da validação e avaliação do produto)																										
Implementação do aplicativo no CHC Avalia TIS Cuidados Paliativos																										
Implementação do aplicativo no Avalia Tis – Paciente Clínico no Hospital																										

de Guarnição de Poto Velho																										
Elaboração Dissertação de Mestrado																										
Defesa Mestrado																										
Elaboração da Versão Final da Tese																										
Defesa da tese de Doutorado																										
Divulgação Resultados																										

CRITÉRIOS PARA SUSPENDER OU ENCERRAR A PESQUISA

A pesquisa poderá ser suspensa, se houver indisponibilidade dos recursos físicos e humanos envolvidos ou de participantes, que inviabilizem sua realização.

O encerramento se dará após a construção e validação da Tecnologia Assistencial de Avaliação Clínica para o Enfermeiro.

LOCAL ONDE SERÁ REALIZADA A PESQUISA

A pesquisa será desenvolvida no Complexo Hospital de Clínicas da Universidade Federal do Paraná, mais especificamente, nas Unidades de: Urgência e Emergência Adulto, Terapia Intensiva Pediátrica, Clínica Médica e Clínica Cirúrgica. Da instituição co-participante, Hospital Infantil Waldemar Monastier, será campo de pesquisa a Unidade de Terapia Intensiva Neonatal.

DEMONSTRATIVO DA EXISTÊNCIA DE INFRA-ESTRUTURA

O projeto de pesquisa foi aprovado pelas unidades, campo de pesquisa, por meio de declaração assinada pela supervisão do serviço médico e de enfermagem.

PROPRIEDADE DAS INFORMAÇÕES

As informações obtidas serão registradas em arquivo eletrônico e, ficarão de posse dos pesquisadores por um período de cinco anos após o termino da pesquisa.

PARTICIPANTES DA PESQUISA:

Os participantes da pesquisa serão majoritariamente adultos do sexo feminino, com idade entre 23 e 60 anos aproximadamente, todos graduados em Enfermagem, que atuam nas unidades campo desta pesquisa. Alguns participantes

poderão ter especialização em áreas a fim e/ou mestrado/doutorado acadêmico ou profissional.

GRUPOS VULNERÁVEIS

Não haverá participantes de grupos vulneráveis nesta pesquisa.

FONTES DE MATERIAL DA PESQUISA

As fontes para obtenção dos dados serão: as bases de dados científicos; livros que tratem do objeto de estudo da pesquisa; informações dos enfermeiros participantes, referente a sua prática clínica; diário de campo das discussões com o grupo de participantes e pesquisadores, sobre a Tecnologia Assistencial proposta.

PREVISÃO DE RESSARCIMENTO DE GASTOS AOS PARTICIPANTES DA PESQUISA

Não haverá nenhum custo financeiro aos participantes nesta pesquisa.

ORÇAMENTO DETALHADO DA PESQUISA

Os materiais previstos para o desenvolvimento da pesquisa se refere aqueles de uso acadêmico, que se utilizará para validação e implementação dos aplicativos para avaliação clínica do paciente.

O custo total aproximado previsto, incluindo a aquisição de materiais e o pagamento do transporte dos pesquisadores, os quais serão de responsabilidade dos pesquisadores e, está descrito na tabela abaixo.

MATERIAL	QUANTIDADE	CUSTO (R$)
Papel sulfite	5000	150,00
Tinta impressora	02	300,00
Pen drive	04	300,00
Notebook	02	7.000,00
Caneta	10	30,00
Pasta	05	50,00
Fita métrica	06	60,00
Esfigmomanômetro	04	600,00
Estetoscópio	04	300,00
Lanterna	04	100,00
Balança Digital	04	500,00
Transporte pesquisadores	Deslocamento campos da pesquisa	2.000,00
**TOTAL**		11.390,00

BIBLIOGRAFIA

ALFARO-LEFEVRe, R. **Aplicação do processo de enfermagem**: fundamentos para o raciocínio clínico-8. ed. - Porto Alegre: Artmed, 2014. Acesso em 20 maio de 2018. Disponível em: https://www.passeidireto.com/arquivo/40069595/alfaro-lefevre-2014-aplicacao-do processodeenfermagem

ALMEIDA, M. A.; LUCENA, A. F.; FRANZEN, E.; LAURENT, M. C.; et al. **Processo de Enfermagem na Prática Clínica: estudos clínicos realizados no Hospital de Clínicas de Porto Alegre.** Porto Alegre: Artmed, 2011.

BENEDET et al, 2016). Processo de enfermagem: instrumento da sistematização da assistência de enfermagem na percepção dos enfermeiros. **res.: fundam. care. online** 2016. jul./set.

BENEDET, S. A.; GELBCKE F. L.; AMANTE, L. N.; et al. Processo de enfermagem: instrumento da sistematização da assistência de enfermagem na percepção dos enfermeiros. **J. res.: fundam. care.** online 2016. jul./set. 8(3): 4780-4788

BOTTOSSO, Rosa Maria. PROCESSO DE ENFERMAGEM NAS ESCOLAS DE NÍVEL TÉCNICO E SUPERIOR DE MATO GROSSO: estudo sobre concepções e práticas educativas docentes,2014. Acesso em 20 maio 2018. Disponível em: www.ufmt.br/ufmt/unidade/userfiles/.../f8e738e6e3d79054e59fb47ab19a206f.pdf

CLAUDINO, H.G; GOUVEIA, E.M.L; SANTOS, S.R; LOPES, E.M.E.L. Auditoria em registros de enfermagem: uma revisão de literatura. **Rev. Enferm. UERJ, 2013**. Acesso em 27 de maio de 2018. Disponível em:

CONSELHO FEDERAL DE ENFERMAGEM (COFEN). Resolução nº 358, de 15 de outubro de 2009. Dispõe sobre a Sistematização da Assistência de Enfermagem e a implementação do Processo de Enfermagem em ambientes, públicos ou privados, em que ocorre o cuidado profissional de enfermagem, e dá outras providências. Portal COFEN, Brasília, DF, 15 out. 2009. Disponível em: http://novo.portalcofen.gov.br/resoluo-cofen-3582009_4384.html. Acesso em: 15 set 2017.

GARCIA, T. R. Sistematização da Assistência de Enfermagem: aspecto substantivo da prática profissional**. Esc Anna Nery** 2016;20(1):5-10.

GARCIA, T. R..; NÓBREGA, M. M. L. Processo de Enfermagem: da teoria à pratica assistência e de pesquisa. **Escola Anna Nery Revista de Enfermagem**, vol. 13, núm.1, janeiro-março, 2009, pp. 188-193. Universidade Federal do Rio de Janeiro. Rio de Janeiro, Brasil.

GIL, A. C. **Métodos e técnicas de pesquisa social.** 6ª ed. – São Paulo: Atlas, 2008.

GUTIÉRREZ, M. G. R.; MORAIS, S. C. R. V. Systematization of nursing care and the formation of professional identity. **Rev Bras Enferm** [Internet]. 2017; 70(2):436-41. DOI: http://dx.doi.org/10.1590/0034-7167-2016-0515.

HORTA, V. de A. Processo de Enfermagem. São Paulo: EPU, 1979.

SANTOS, Z. M. S. A. **Tecnologias em saúde**: da abordagem teórica a construção e aplicação no cenário do cuidado [livro eletrônico]. – Fortaleza: EDUECE, 2016.

SILVA, R. C.; FERREIRA, M. A. **Tecnologia no cuidado de enfermagem**: uma análise a partir do marco conceitual da Enfermagem Fundamental. Rev Bras Enferm. 2014 jan-fev; 67(1): 111-8.
