# Supplementary material for: Evaluation of software technical quality for collecting data from patients under palliative care
Source: Rev Bras Enferm. 2024 Jul 29;77(3):e20230435. doi: 10.1590/0034-7167-2023-0435 (PMC11290725; doi:10.1590/0034-7167-2023-0435)
Supplement: Supplementary file 2 [file 0034-7167-reben-77-03-e20230435-suppl02.pdf]

Tabela 7 - Distribuição dos valores de acordo com as subcaracterísticas e características de qualidade do Avalia Tis - Cuidados Paliativos pelos enfermeiros e especialistas em TI (n=16)

Variável	Enfermeiro	TI
	n = 8	n = 8
	VC (%)	VC (%)
Integridade Funcional	100,0	93,8
Correção Funcional	95,8	83,3
Aptidão Funcional	87,5	75,0
Adequação funcional	94,4	84,0
Maturidade	100,0	62,5*
Tolerância a Falhas	100,0	87,5
Recuperabilidade	100,0	42,9*
Disponibilidade	100,0	87,5
Confiabilidade	100,0	70,1
Reconhecimento de adequação	93,8	65,6*
Apreensibilidade	95,8	70,8
Operabilidade	100,0	75,0
Acessibilidade	50,0	33,3*
Proteção contra Erro	100,0	87,5
Estética de interface do usuário	100,0	68,8*
Usabilidade	89,9	66,8*
Tempo	100,0	87,5
Recursos	87,5	87,5
Capacidade	100,0	83,3
Eficiência de Desempenho	95,8	86,1
Interoperabilidade	91,7	79,2
Coexistência	100,0	80,0
Compatibilidade	95,8	79,6
Confidencialidade	100,0	87,5
Integridade	80,0	86,6
Não repúdio	100,0	85,7
Responsabilização	100,0	71,4
Autenticação	100,0	85,7
Segurança	96,0	83,4

Fonte: Autores (2023)

Legenda: VC - Valor Medido da Característica; % - valor em percentual;

TI - Especialistas em Tecnologia da informação

* valor de VC < 70%
