# Supplementary material for: Evaluation of software technical quality for collecting data from patients under palliative care
Source: Rev Bras Enferm. 2024 Jul 29;77(3):e20230435. doi: 10.1590/0034-7167-2023-0435 (PMC11290725; doi:10.1590/0034-7167-2023-0435)
Supplement: Translation

# Translation: Avaliação da qualidade técnica de *software* para coleta de dados de pacientes em cuidados paliativos

**DOI:** 10.1590/0034-7167-2023-0435pt

**Authors:** Jéssica de Fátima Gomes Pereira, Letícia Pontes, Mitzy Tannia Reichembach Danski, Solena Ziemer Kusma Fidalski, Juliana de Oliveira dos Santos, Maria Gorete de Brito Cunha, Mairla Cristina Silva Mota, Edmilson Bezerra Cruz

**Affiliations:** I Universidade Federal do Paraná, Complexo Hospital de Clínicas, Empresa Brasileira de Serviços Hospitalares. Curitiba, Paraná, Brasil; II Universidade Federal do Paraná. Curitiba, Paraná, Brasil

**Keywords:** Cuidados Paliativos, Software, Coleta de Dados, Processo de Enfermagem, Avaliação de Tecnologia Biomédica

## Abstract

**Objetivos:**

avaliar a qualidade técnica de *software* para coleta de dados de pacientes sob cuidados paliativos.

**Métodos:**

trata-se de pesquisa metodológica de avaliação de tecnologia, conforme norma técnica *International Organization for Standardization/International Electrotechnical Comission* 25040-2011, desenvolvida de agosto de 2021 a agosto de 2023. Participaram oito enfermeiros e oito profissionais de tecnologia da informação como juízes, os quais avaliaram seis características e 23 subcaracterísticas de qualidade. Consideraram-se adequados os itens que atingiram porcentual de concordância superior a 70%.

**Resultados:**

as características avaliadas pelos enfermeiros/profissionais de tecnologia da informação receberam os seguintes porcentuais de concordância, respectivamente: adequação funcional (94%-84%); confiabilidade (100-70%); usabilidade (89,9-66,8%); eficiência de desempenho (95,8%-86,1%); compatibilidade (95,8-79,6%); e segurança (96%-83,4%).

**Conclusões:**

o *software* foi considerado adequado na avaliação de qualidade para oferecer suporte ao enfermeiro na coleta de dados do paciente em cuidados paliativos, com potencial para operacionalizar a primeira etapa do Processo de Enfermagem.

## INTRODUÇÃO

A Organização Mundial da Saúde (OMS) define Cuidados Paliativos (CP) como abordagem que melhora a qualidade de vida de pacientes, adultos e crianças que experienciam problemas associados a doenças potencialmente fatais. Esses cuidados, por sua vez, envolvem pacientes e família e se caracterizam por ações que buscam integrar aspectos emocionais, sociais, espirituais e culturais^(1)^.

Ao considerar a dimensão desses cuidados, determinar como o enfermeiro deve assistir o paciente em CP é complexo e desafiador, pois as ações de cuidado devem estar amparadas em instrumento metodológico de trabalho, como o Processo de Enfermagem (PE), o qual permitirá avaliação ampla e sistematizada do cuidado. Nos CP, a primeira etapa do PE está relacionada, especialmente, à identificação de sinais e sintomas. Logo, a coleta de dados é a base para elaboração de um plano de cuidado individualizado e eficaz que garante conforto e morte digna^(2)^.

Para melhorar a coleta de dados em CP, em especial na identificação e no manejo dos sinais e sintomas, os profissionais de saúde podem utilizar tecnologias, como as Tecnologias de Informação e Comunicação (TIC). Na área da saúde, as TIC tornaram-se relevantes estratégias na gestão do conhecimento, principalmente pela digitalização das informações. Dessa forma, profissionais de saúde incorporam esse tipo de tecnologia para melhorar a assistência e a produção de pesquisas científicas^(3)^.

Como exemplo de TIC utilizadas na área da saúde, tem-se os aplicativos para dispositivos móveis, que dão suporte a pacientes e profissionais de saúde na realização do cuidado. Como exemplo, citam-se o aplicativo *point-of-care*, desenvolvido na Austrália para promover adesão às recomendações de boas práticas para o gerenciamento de sintomas de distúrbios neurocognitivos^(4)^, o *NeMo system*, ferramenta móvel para identificação domiciliar de doenças neonatais em Uganda^(5)^, e aplicativo Meu PICC, desenvolvido em São Paulo para o acompanhamento extra-hospitalar de pacientes em uso de cateter central de inserção periférica^(6)^.

Contudo, ao observar na prática clínica a necessidade de uma tecnologia para subsidiar a operacionalização da primeira etapa do PE, ou seja, a coleta de dados de pacientes em CP, entre os anos de 2017 e 2019, foi desenvolvido um *software* do tipo aplicativo móvel denominado AVALIA TIS - Cuidados Paliativos, que tem como finalidade subsidiar a realização da coleta de dados de pacientes em CP. Esse aplicativo é fundamentado na Teoria das Necessidades Humanas Básicas e na filosofia paliativista, que busca o conforto como objetivo do cuidado^(7)^.

Todavia, considera-se que, para segurança na utilização de um *software*, faz-se necessário avaliar a qualidade, que é definida por normativas com especificações técnicas que definem regras e critérios para assegurar que os produtos de *software* estejam adequados para o devido uso. Essas normas são essenciais, pois direcionam o desenvolvimento e a avaliação dos produtos de *software* com o objetivo de atender à expectativa e qualidade exigida pelo cliente^(8)^. Dessa forma, para dar continuidade à pesquisa que resultou no desenvolvimento do AVALIA TIS - Cuidados Paliativos, optou-se por desenvolver estudo de avaliação de qualidade técnica para essa tecnologia móvel.

Entende-se que, para garantir a segurança na utilização de um *software*, é necessária avaliação da qualidade técnica para identificar se a tecnologia atende às necessidades dos usuários a curto, médio e longo prazo, além de prover eficiência e segurança na utilização, com custos, produtividade e funcionalidade adequados, permitindo o uso com poucos ou nenhum defeito^(9)^.

Ressalta-se que o AVALIA TIS - Cuidados Paliativos é produto de dissertação de mestrado profissional em enfermagem da Universidade Federal do Paraná (UFPR) para subsidiar enfermeiros na coleta de dados de pacientes sob CP. O aplicativo também fortalece o registro da primeira etapa do PE, ao organizar os dados do paciente de forma sistematizada em documento no formato *Portable Document Format* (PDF), o qual pode ser anexado em prontuário^(7)^.

## OBJETIVOS

Avaliar a qualidade técnica do aplicativo móvel AVALIA TIS - Cuidados Paliativos, desenvolvido para subsidiar enfermeiros na coleta de dados de pacientes sob CP.

## MÉTODOS

### Aspectos éticos

Esta pesquisa é vinculada ao projeto temático intitulado “Tecnologias para Qualificar e Consolidar a Sistematização da Assistência de Enfermagem nos Diferentes Cenários da Prática Profissional”, aprovado pelo Comitê de Ética em Pesquisa (CEP) do Complexo Hospital de Clínicas da UFPR (CHC-UFPR). Foi solicitada a assinatura do Termo de Consentimento Livre e Esclarecido (TCLE) para os juízes que participaram da avaliação da qualidade do *software*.

### Desenho, local do estudo e período

Trata-se de pesquisa metodológica de avaliação de tecnologia, conforme norma técnica *International Organization for Standardization* (ISO)*/International Electrotechnical Comission* (IEC) 25040-2011^(10)^. A pesquisa foi desenvolvida através do Programa de Pós-Graduação em Enfermagem da UFPR, em Curitiba, Paraná, Brasil, de agosto de 2021 a agosto de 2023.

### População ou amostra; critérios de inclusão e exclusão

Obtiveram-se dois grupos de juízes constituídos por enfermeiros e profissionais de tecnologia da informação (TI) para avaliação do *software*. Para definição da amostra, utilizaram-se as recomendações da norma ISO*/*IEC- 25062, que indica amostragem mínima de oito avaliadores para garantir a representatividade da amostra^(11)^.

A seleção dos profissionais para compor o grupo de juízes ocorreu por meio da técnica bola de neve (*snowball sampling*)^(12)^. Os critérios de inclusão dos avaliadores foram ser enfermeiro com experiência acadêmica e/ou prática em CP, ser profissional da área de TI com experiência acadêmica e/ou prática em desenvolvimento de aplicativos móveis. O critério de exclusão foi ser avaliadores que não responderam ao instrumento no tempo determinado.

Os juízes foram classificados quanto ao nível de *expertise*. Nessa classificação, o grau de conhecimento é dividido em cinco níveis, que vão do *novice* ao *expert*, descritos na Figura 1 ^(13)^.

**Figura 1:** Classificados quanto ao nível de *expertise*, Curitiba, Paraná, Brasil, 2023

*Nota: adaptado de BENNER; TANNER; CHESLA (2009).*

Para o estabelecimento do nível de *expertise*, utilizou-se a fórmula N = (X + Y + Z)/3, em que X corresponde ao tempo de prática, Y, ao tempo de grupo de pesquisa, e Z, ao conhecimento científico (titulação, trabalho de titulação e produção científica na área de CP) (Figura 2)^(13)^. Ressalta-se que a classificação foi realizada para fins de nivelamento, não sendo utilizada como critério de inclusão e exclusão.

**Figura 2:** Classificação conforme o nível de experiência prática e acadêmica, Curitiba, Paraná, Brasil, 2023

*Nota: adaptado de BENNER; TANNER; CHESLA (2009).*

### Protocolo do estudo

Para avaliar o *software*, adotou-se o processo de avaliação da qualidade de *software* da norma ISO/IEC 25040-2011, que consiste em cinco etapas^(10)^:

#### Etapa I - Estabelecer os requisitos da avaliação

Para instituir os requisitos de avaliação do *software*, optou-se pelo Modelo de Qualidade de Produto, definido pela norma ISO/IEC 25010-2011, o qual estabelece oito características e respectivas subcaracterísticas que devem ser contempladas na avaliação: adequação funcional; eficiência de desempenho; compatibilidade; usabilidade; confiabilidade; segurança; manutenibilidade; e portabilidade^(14)^. Ressalta-se que, devido à impossibilidade de disponibilização do código fonte do *software* aos avaliadores, por motivo de segurança da tecnologia, determinou-se a avaliação de seis das oito características de qualidade, excluindo-se a manutenibilidade e a portabilidade (Figura 3).

**Figura 3:** Modelo de Qualidade de Produto: características e subcaracterísticas, Curitiba, Paraná, Brasil, 2023

*Nota: adaptado da ABNT ISO/IEC 25010 (2011); tradução livre da autora.*

#### Etapa II - Especificar a avaliação

Nessa etapa, definiram-se as métricas de qualidade, os níveis de pontuação e os critérios para julgamento. No processo de avaliação de qualidade, utilizaram-se dois formulários para coleta de dados referentes ao *software*; esses formulários já foram validados quanto à clareza, compreensão e objetividade em estudo anterior^(15)^. Para realização desta pesquisa, os formulários foram adaptados e enviados para o grupo de enfermeiros e para o grupo de especialistas em TI, contendo questões referentes a dados de identificação e informações relacionadas às características e subcaracterísticas de qualidade. Esses instrumentos tiveram níveis de pontuação com base na ABNT NBR ISO/IEC 14598-6 Anexo C: (C) Concordo; (D) Discordo; (NA) Não se Aplica; e Comentários. O nível C significa que o item atende ao requisito de qualidade; o nível D significa que o item não atende ao requisito de qualidade; e o nível NA é referente ao item não se aplica ou não foi avaliado^(8)^.

As questões que os avaliadores não conseguiram avaliar, seja por ausência de informação, recursos ou conhecimento específico, assinaladas com a opção “NA”, foram descartadas. Essas respostas não pontuam e, portanto, não geram prejuízo na avaliação.

Os valores em porcentuais das características de qualidade avaliadas foram obtidos pela fórmula matemática descrita na Figura 4.

**Figura 4:** Fórmula para o cálculo de porcentuais das características e subcaracterísticas de qualidade, Curitiba, Paraná, Brasil, 2023

*Nota: adaptado de Oliveira e Peres (2015).*

Consideraram-se adequados os itens com porcentual de concordância maior do que 70%, conforme a escala de avaliação para as subcaracterísticas, adaptada a partir da norma técnica da ABNT NBR ISO/IEC 14598-6 Anexo C (Informativo) (2004), em que 25% refere-se a fraco, 50%, a regular, 75%, a bom, e 100%, a excelente^(8,15)^.

#### Etapa III - Projetar e planejar as atividades de avaliação

Essa etapa caracterizou-se pelo planejamento das atividades e pela seleção dos participantes para avaliação do *software*. A coleta de dados ocorreu por meio eletrônico, com o envio de carta-convite para os possíveis participantes da pesquisa, no período de setembro de 2022 a janeiro de 2023. Àqueles que concordaram em participar da pesquisa, solicitou-se leitura e assinatura do TCLE. Esses também receberam informações sobre o aplicativo e o processo de avaliação das características de qualidade a serem avaliadas, além do *link* para efetuar o *download* do aplicativo e o *link* para acesso ao formulário de avaliação da qualidade do *software* do *Google Forms* ^®^. O aplicativo teste enviado para os participantes apresenta especificações técnicas compatíveis apenas com o sistema Android e, portanto, não pode ser utilizado em dispositivos com sistema iOS.

Os participantes tiveram prazo de dez dias para devolução do formulário preenchido, no entanto foi necessário estender o prazo por mais dez dias, a fim de garantir que os avaliadores finalizassem o processo. Durante esse período, a pesquisadora esteve disponível para esclarecer as dúvidas e auxiliar os participantes via e-mail, telefone e *WhatsApp* ^®^.

#### Etapa IV - Executar a avaliação

Os dados foram coletados e tabulados em planilhas do *Microsoft Excel* ^®^ e analisados pelo programa computacional *Statistical Package for the Social Sciences* (SPSS^®^) (IBM^®^ SPSS^®^ *Statistics* v. 25.0, SPSS Inc, Chicago, EUA). Os resultados foram expressos por médias, medianas, valores mínimos, valores máximos e desvios padrão (variáveis quantitativas) ou por frequências e porcentuais (variáveis qualitativas). Para efetivação dessa etapa, optou-se pela ajuda de uma estatística.

#### Etapa V - Concluir a avaliação

A análise dos dados teve como finalidade concluir se o AVALIA TIS - Cuidados Paliativos apresenta qualidade técnica para a maioria das características e subcaracteristicas avaliadas. Os dados estão apresentados no formato de tabelas.

## RESULTADOS

Participaram 16 juízes, sendo oito enfermeiros e oito profissionais de TI. Entre os enfermeiros, prevaleceu o sexo feminino, com sete mulheres (87,5%) e um homem (12,5%) com idades entre 28 e 62 anos. Em relação à titulação, cinco (62,5%) eram mestres e três (37,5%) eram especialistas. Quanto ao nível de *expertise*, três foram classificados (37,5%) como *novice*, três, (37,5%) como *advenced beginner*, e dois (25%), como *competent*.

Em relação aos juízes profissionais de TI, a maioria era do sexo masculino, seis homens (75%) e duas mulheres (25%), com idades 23 e 46 anos. Desses, seis (75%) eram especialistas e dois (25%) eram doutores. O nível de *expertise* foi classificado em cinco (62,5%) *novice*, um (12,5%) *advenced beginner*, um (12,5%) *competent* e um (12,5%) *proficient*.

A técnica bola de neve para seleção dos juízes permitiu a avaliação do aplicativo por profissionais de quatro regiões do Brasil (Sul, Sudeste, Norte, Nordeste) e de um juiz de Portugal, permitindo avaliação da tecnologia por diferentes visões. Os resultados da avaliação do *software*, por meio dos instrumentos utilizados, estão apresentados na Tabela 1.

**Tabela 1:** Distribuição dos valores de acordo com as subcaracterísticas e características de qualidade do AVALIA TIS - Cuidados Paliativos pelos enfermeiros e especialistas em tecnologia da informação, Curitiba, Paraná, Brasil, 2023 (N=16)

Variáveis	Enfermeiro	TI
	n = 8	n = 8
	VC (%)	VC (%)
Integridade funcional	100,0	93,8
Correção funcional	95,8	83,3
Aptidão funcional	87,5	75,0
Adequação funcional Maturidade	94,4 100,0	84,0 62,5^*^
Tolerância a falhas	100,0	87,5
Recuperabilidade	100,0	42,9^*^
Disponibilidade	100,0	87,5
Confiabilidade Reconhecimento de adequação	100,0 93,8	70,1 65,6^*^
Apreensibilidade	95,8	70,8
Operabilidade	100,0	75,0
Acessibilidade	50,0	33,3^*^
Proteção contra erro	100,0	87,5
Estética de interface do usuário	100,0	68,8^*^
Usabilidade Tempo	89,9 100,0	66,8^*^ 87,5
Recursos	87,5	87,5
Capacidade	100,0	83,3
Eficiência de desempenho Interoperabilidade	95,8 91,7	86,1 79,2
Coexistência	100,0	80,0
Compatibilidade Confidencialidade	95,8 100,0	79,6 87,5
Integridade	80,0	86,6
Não repúdio	100,0	85,7
Responsabilização	100,0	71,4
Autenticação	100,0	85,7
Segurança	96,0	83,4

VC - valor medido da característica; % - valor em porcentual; TI - especialistas em tecnologia da informação;

* valor de VC < 70%.

Na avaliação da adequação funcional, observou-se que, na subcaracterística integridade funcional, todos os juízes enfermeiros concordaram que o aplicativo atende à capacidade de subsidiar a avaliação do paciente em CP. Já na característica confiabilidade, as subcaracterísticas maturidade e recuperabilidade obtiveram avaliação abaixo de 70% pelos juízes da TI. Esses sugeriram melhorar a capacidade do aplicativo de salvar as informações cadastradas antes de finalizar o processo de coleta de dados. Um dos juízes, codificado com a letra T, seguida de numeração, na avaliação dessa característica, alertou que o aplicativo:

*Não possui rotina de retomada de preenchimento no caso de interrupção do processo.* (T1) - (recuperabilidade)

A característica usabilidade foi a única característica que apresentou a média de avaliação abaixo de 70% pelos juízes da TI. Para as subcaracterísticas reconhecimento de adequação, apreensibilidade, proteção contra erro e estética de interfaces do usuário, os juízes apontaram que:

*Nas fases do questionário, sempre no início, há breve explicação sobre o objetivo do questionário. Há escala sobre as dores e o que cada nível da escala representa.* (T4) - (reconhecimento e adequação)

*Campos com escalas devem possuir valores sugeridos de preenchimento, e a navegação entre os campos se dá apenas pela seleção do campo.* (T1) - (apreensibilidade)

*Nos campos em que precisa entrar dados que sejam números, ele não deixa entrar letras.* (T5) - (proteção contra erro)

*Tem muito potencial para melhorar. Acretido que o menu ficaria melhor se estivesse centralizado.* (T5) - (estética de interfaces)

A característica usabilidade também recebeu comentários dos juízes enfermeiros, em especial nas subcaracterísticas reconhecimento de adequação, acessibilidade e estética de interface do usuário. Os juízes enfermeiros foram codificados com a letra E, seguida de numeração:

*Aplicativo é autoexplicativo.* (E5) - (reconhecimento de adequação)

*Não consegui identificar suporte para uso de pessoas com deficiência.* (E5) - (acessibilidade)

*Poderia ser oferecida a possibilidade de alterar a cor se o usuário desejar.* (E3) - (estética de interfaces)

Nas características eficiência de desempenho e compatibilidade, todas as subcaracterísticas receberam avaliação acima de 70%, com destaque para o tempo de resposta e a interoperabilidade do AVALIA TIS - Cuidados Paliativos:

*Seu tempo de resposta ao toque foi muito bom.* (T1) - (tempo)

*Capacidade de armazenamento adequado.* (T7) - (capacidade)

*A interação é boa para trocar informações de maneira eficaz e eficiente.* (T7) - (interoperabilidade)

*O tempo é um pouco longo no primeiro acesso.* (E3) - (tempo)

*Avaliação de enfermagem traz um resumo de tudo que foi avaliada nos módulos do aplicativo.* (E5) - (interoperabilidade)

A sexta e última característica avaliada, segurança, teve quatro subcaracterísticas avaliadas com porcentual acima de 70% por ambos os grupos de juízes, que consideram que o aplicativo é seguro para uso na prática clínica:

*Tentei entrar com outro e-mail, e não deixou.* (T5) - (integridade)

*Necessita preenchimento do cadastro, inclusive com identificação do número do COREN e autorização para uso do aplicativo.* (E5) - (autenticação)

## DISCUSSÃO

O uso de tecnologias assistenciais para subsidiar a operacionalização do PE é um fato, estando em vasto desenvolvimento na realidade brasileira, o que indica progressiva informatização da enfermagem. A utilização desses recursos digitais como suporte para o enfermeiro serve para potencializar, qualificar e efetivar a realização do cuidado^(16)^.

Neste estudo, avaliaram-se seis características e 23 subcaracterísticas referentes à qualidade do aplicativo AVALIA TIS - Cuidados Paliativos. Das seis características avaliadas, apenas a característica usabilidade apresentou porcentual de avaliação abaixo de 70% pelos juízes profissionais da TI, fato que não inviabilizou a avaliação dos juízes enfermeiros, que consideraram essa característica adequada.

Os juízes enfermeiros consideraram que o aplicativo é 89,9% adequado, e isso reflete a capacidade do aplicativo em dar suporte para o enfermeiro realizar a coleta de dados específica para o paciente em CP. Os juízes também consideram que o aplicativo é autoexplicativo, fato que torna uma tecnologia de fácil manuseio pelos usuários.

Contudo, é possível observar algumas sugestões para aprimorar a usabilidade do aplicativo, como a necessidade de menu mais dinâmico para facilitar a avaliação do paciente. Destaca-se que esse aspecto foi planejado no início do desenvolvimento da tecnologia, porém, por limitações financeiras, algumas especificações não foram contempladas na primeira versão. Ainda, na característica usabilidade, a subcaracteristica acessibilidade recebeu pontuação abaixo da média esperada. Esse aspecto não foi pensado no desenvolvimento do AVALIA TIS - Cuidados Paliativos, sendo uma subcaracterística que deve ser pensada em uma próxima versão do aplicativo.

Como benefícios evidenciados na avaliação do AVALIA TIS - Cuidados Paliativos, observou-se que esse *software* apresenta facilidade de acesso, por se tratar de aplicativo móvel que permite o enfermeiro avaliar o paciente à beira do leito. Entende-se que, com o avanço dos telefones celulares, o escopo dos aplicativos para *smartphones* se expandiu, sendo crescente o número de aplicativos desenvolvidos para uso na avaliação clínica de pacientes^(17)^.

O uso de aplicativos de alta qualidade na área da saúde pode ser responsável por agrupar informações necessárias para o surgimento de novas descobertas para a prática clínica, além de interligar informações para melhorar a assistência e modificar radicalmente o atendimento ao paciente^(3)^. Nesse contexto, na Suíça, em 2019, foi desenvolvido e avaliado um aplicativo para subsidiar os cuidados de enfermagem à beira do leito. Esse aplicativo possui diversas funcionalidades que permitem o profissional identificar os dados do paciente, como possibilitar o acesso ao prontuário através da leitura de código presente nas pulseiras de identificação dos pacientes utilizando a câmera do *smartphone* ^(18)^.

A característica eficiência de desempenho do AVALIA TIS - Cuidados Paliativos obteve boa avaliação dos juízes, com destaque para o tempo de resposta do aplicativo e da capacidade de armazenamento. Como a ideia central da tecnologia é oferecer ao enfermeiro suporte para realização de avaliação clínica robusta que atenda a todas as dimensões do paciente em CP, o aplicativo é extenso, sendo necessários alguns acessos para o usuário se adaptar às interfaces.

Para realizar a avaliação clínica do paciente em CP, é preciso entender que esses cuidados foram criados para tratar a pessoa como um todo e não apenas a doença. Dessa forma, a priorização de domínios de funcionamento tipicamente periféricos, como psicossocial, espiritual e existencial, torna-se fundamental. O objetivo geral desses cuidados é a obtenção da melhor qualidade de vida possível^(19)^.

No contexto dos aplicativos móveis utilizados na saúde, o AVALIA TIS - Cuidados Paliativos tem funcionalidade semelhante ao aplicativo CuidarTech Semio - Exame Clínico de Enfermagem, pois, além de ser uma inovação tecnológica, ambos são sustentados pela Teoria das Necessidades Humanas Básica, servindo como ferramenta de apoio educacional e profissional que ajuda estudantes e enfermeiros na realização da coleta de dados, favorecendo o conhecimento científico e o raciocínio clínico^(20)^.

Outro benefício encontrado pelos juízes na avaliação de qualidade técnica foi a característica interoperabilidade, pela capacidade do aplicativo gerar resumo de todos os dados coletados na avaliação clínica, garantindo o registro da primeira etapa do PE. Há que se considerar que o registro dos dados colhidos pelo enfermeiro no prontuário é uma forma de comunicação multiprofissional. Esse registro pode ser realizado em meio tradicional ou eletrônico, garantindo a continuidade e a qualidade da assistência^(21)^.

Além do registro gerado, a segurança do aplicativo é ponto de destaque na avaliação de qualidade, envolvendo a confidencialidade dos dados inseridos. Para acesso aos dados, o *software* exige do profissional um cadastro, aprovado pelo administrador do aplicativo, que pode ser o responsável pela unidade onde a tecnologia será utilizada. O aplicativo também permite que o usuário possa recuperar a senha de acesso, o que facilita a utilização da tecnologia.

Considera-se que o processo de avaliação da qualidade técnica utilizada neste estudo é complexo, visto que envolve uma série de fases e etapas para que as características de qualidades sejam avaliadas e permitam apontar se a tecnologia é capaz de suprir a necessidade do usuário. Os benefícios encontrados na avaliação de qualidade técnica do AVALIA TIS - Cuidados Paliativos apontam que essa é uma tecnologia inovadora, pois é capaz de guiar o enfermeiro a realizar uma coleta de dados detalhada do paciente em CP, possuindo uma série de escalas específicas para mensuração de sinais e sintomas. Essa tecnologia é de fácil acesso, possibilitando o enfermeiro a ter um resumo detalhado e fidedigno dos dados coletados e dando subsídios para a realização de um plano de cuidados direcionado às reais necessidades do paciente.

### Limitações do estudo

Esta pesquisa apresentou limitações relacionadas ao aplicativo teste, por ser compatível apenas com o sistema Android, situação que dificultou o aceite dos juízes para realizar a coleta de dados, além da necessidade de uma unidade de atendimento a pacientes em CP para aplicação da tecnologia na prática clínica.

### Contribuições para a área da enfermagem

O processo de avaliação da qualidade técnica utilizado neste estudo evidenciou que o AVALIA TIS - Cuidados Paliativos é uma ferramenta com potencial para ser empregada tanto para subsidiar o enfermeiro na prática clínica quanto para o ensino na saúde, com o intuito de direcionar o olhar do graduando à multidimensionalidade do indivíduo, além de ser um instrumento para coleta de dados em futuras pesquisas. Ainda, entende-se que o método utilizado nesta pesquisa pode servir de base para futuros estudos que apresentem como tema a avaliação da qualidade técnica de *software* na saúde. Considera-se que esta pesquisa é um avanço para a área da enfermagem no que se refere à utilização segura de *software* para a prática do cuidado.

## CONCLUSÕES

A qualidade do aplicativo AVALIA TIS - Cuidados Paliativos mostra-se adequada para avaliação multidimensional do paciente em CP, possibilitando ao enfermeiro avançar nas demais etapas do PE. Ao considerar que o processo de avaliação da qualidade do AVALIA TIS - Cuidados Paliativos envolveu uma série de características, essa tecnologia móvel apresentou bons resultados, apesar da avaliação abaixo da média na característica usabilidade pelo grupo de juízes da TI.

Os comentários registrados mostram que a primeira versão do aplicativo é de fácil acesso para o enfermeiro. No entanto, pode ter investimentos para melhorias, como a inserção de um menu que permita o enfermeiro selecionar a *interface* de avaliação que deseja, sem necessidade de passar por todas as telas. O objetivo geral do aplicativo, a avaliação clínica, é reconhecida na avaliação dos juízes, com destaque para o registro após o processo de avaliação, benefício que garante a finalização da primeira etapa do PE.

Como sugestão para pesquisas futuras, recomenda-se que o AVALIA TIS - Cuidados Paliativos possa ser avaliado na prática clínica de enfermeiros e por estudantes de enfermagem, a fim de verificar sua eficácia para avaliação de aspectos relevantes do paciente em CP.

## Data Availability

https://doi.org/10.48331/scielodata.CANHHY
